# Oestrogen Receptor-α binds the *FOXP3* promoter and modulates regulatory T-cell function in human cervical cancer

**DOI:** 10.1038/s41598-017-17102-w

**Published:** 2017-12-11

**Authors:** Sreenivas Adurthi, Mahesh M. Kumar, H. S. Vinodkumar, Geetashree Mukherjee, H. Krishnamurthy, K. Kshitish Acharya, U. D. Bafna, Devi K. Uma, B. Abhishekh, Sudhir Krishna, A. Parchure, Murali Alka, R. S. Jayshree

**Affiliations:** 1 Department of Microbiology, Kidwai Memorial Institute of Oncology, Bangalore, India; 2 Shodhaka Life Sciences Private Limited, Bangalore, India; 3 Department of Pathology, Kidwai Memorial Institute of Oncology, Bangalore, India; 4 National Center for Biological Sciences, TIFR, Bangalore, India; 5 Institute of Bioinformatics And Applied Biotechnology, Bangalore, India; 6 Department of Gynecology, Kidwai Memorial Institute of Oncology, Bangalore, India; 7 Department of Immunohematology, Kidwai Memorial Institute of Oncology, Bangalore, India; 8 Present Address: Structural Biology Lab, School of Bio Sciences and Technology, Vellore Institute of Technology, Vellore, India; 9 Present Address: Department of Histopathology, Tata Medical Center, Kolkata, India; 10 Present Address: Department of Transfusion Medicine, JIPMER, Puducherry, India

## Abstract

Oestrogen controls *Foxp3* expression in regulatory T cells (T_reg_ cells) via a mechanism thought to involve oestrogen receptor alpha (ERα), but the molecular basis and functional impact of ERα signalling in T_reg_ cells remain unclear. We report that ERα ligand oestradiol (E2) is significantly increased in human cervical cancer (CxCa) tissues and tumour-infiltrating T_reg_ cells (CD4^+^CD25^hi^CD127^low^), whereas blocking ERα with the antagonist ICI *182,780* abolishes *FOXP3* expression and impairs the function of CxCa infiltrating T_reg_ cells. Using a novel approach of co-immunoprecipitation with antibodies to E2 for capture, we identified binding of E2:ERα complexes to FOXP3 protein in CxCa-derived T_reg_ cells. Chromatin immunoprecipitation analyses of male blood T_reg_ cells revealed ERα occupancy at the *FOXP3* promoter and conserved non-coding DNA elements 2 and 3. Accordingly, computational analyses of the enriched regions uncovered eight putative oestrogen response elements predicted to form a loop that can activate the *FOXP3* promoter. Together, these data suggest that E2-mediated ERα signalling is critical for the sustenance of *FOXP3* expression and T_reg_ cell function in human CxCa via direct interaction of ERα with *FOXP3* promoter. Overall, our work gives a molecular insight into *ERα* signalling and highlights a fundamental role of E2 in controlling human T_reg_ cell physiology.

## Introduction

Regulatory T cells (T_reg_ cells) expressing forkhead box P3 (*FOXP3*) critically control immune responses by maintaining tolerance of self-antigens and restricting inflammation. T_reg_ cells are indispensable for host protection against autoimmunity, allergy and inflammatory tissue damage, but these cells can also exert detrimental effects by mediating inappropriate tolerance of infections and tumours^1^. FOXP3 is a lineage-specific transcription factor that governs T_reg_ development, differentiation, maintenance and function^2^. While a major proportion of T_reg_ cells differentiates in the thymus (tT_reg_ cells), a small percentage arises in the periphery (pT_reg_ cells), with both subsets being evenly distributed between the lymphoid and non-lymphoid organs, as well as in the tissue-resident T_reg_ cell pool^3^. Signals in the local microenvironment stimulate tissue-resident T_reg_ cells to undergo phenotypic and functional specialization tailored to specific anatomic sites and organs^3^. These environment-specific cues include cytokines such as IL-2 and TGFβ, hormones such as oestradiol (E2) and 1,25-Dihyroxyvitamin D3, vitamin metabolites such as retinoic acid, and even microbial products such as short-chain fatty acids^4,5^. Indeed, sex steroid hormone E2 has previously been shown to drive T_reg_ cell expansion and induce/potentiate their suppressive functions by signalling through oestrogen receptor alpha (ERα), thereby enhancing the phosphorylation of Akt and/or increasing cell surface expression of the inhibitory receptor PD-1^6–12^. While these data have convincingly demonstrated that oestradiol can potently modulate T_reg_ cell function, the molecular basis of E2 signalling in T_reg_ cells and how this impacts on immune surveillance in the human female genital tract remains largely unknown largely because in *ERα (Esr1)* knockout mice *Foxp3* expression was retained^6^.

In the present study, we demonstrate that human cervical cancer (CxCa) and tumour-infiltrating T_reg_ cells (CD4+CD25^hi^CD127^low^ cells) contain elevated levels of hormone E2 which is in complex with ERα in the latter. Using the ER-specific antagonist ICI *182,780* (ICI), we further show that ERα modulates *FOXP3* expression and suppressive function of T_reg_ cells isolated from CxCa tumour tissues. Using a novel approach of immunoblotting of E2-bound proteins revealed that ERα can form complexes with FOXP3 protein. Further, analysis in male blood T_reg_ cells by chromatin immunoprecipitation (ChIP)-coupled quantitative PCR (qPCR) demonstrated ERα occupancy of the *FOXP3* promoter and multiple intronic enhancers, consistent with an ability of ERα to directly modulate *FOXP3* gene expression. Accordingly, computational analyses of the enriched regions of the *FOXP3* locus identified eight putative oestrogen responsive elements (ERE) predicted to form a loop that may be capable of activating the *FOXP3* promoter. Taken together, these data reveal a novel role of E2-mediated ERα signalling in the transcriptional regulation of *FOXP3* and control of human T_reg_ cell function.

## Results

### Human cervical tumours display accumulation of sex steroid hormone oestradiol

**Figure 1:**
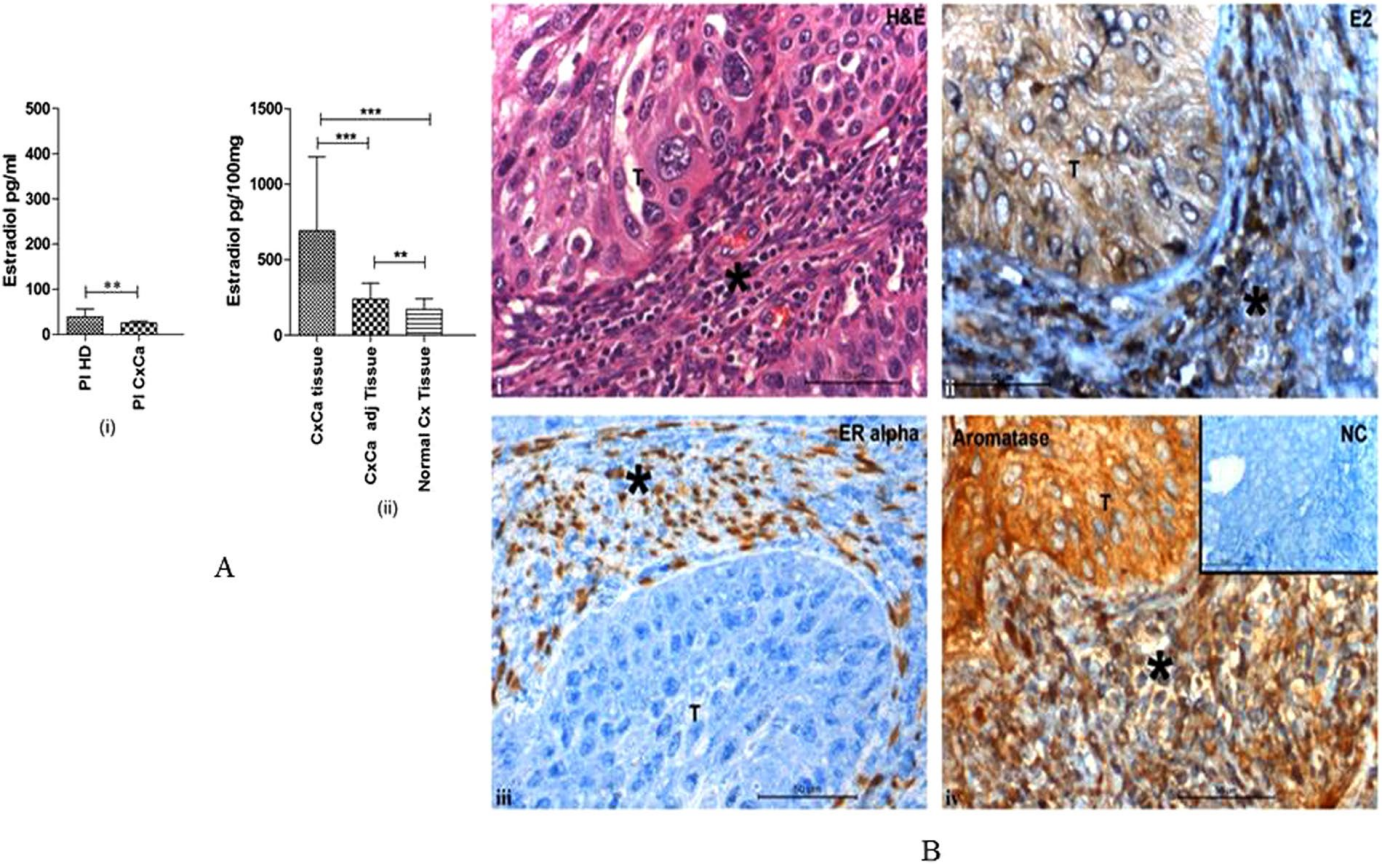
Cervical tumours are enriched in oestradiol (E2) and express oestrogen receptor α. (**A**) (i) Concentrations of 17β-oestradiol as determined by ELISA in blood plasma from healthy donors (Pl HD) or patients with CxCa (Pl CxCa) as well as in (ii) tissue samples of cervical tumours (CxCa), areas adjacent to the tumours (CxCa adj), and healthy cervices (Normal Cx). Graph shows mean values ± SEM of n = 30 per group. (**B**) Staining distribution of 17β oestradiol, oestrogen receptor α, and aromatase in a representative tissue section of SCC cervix. Upper left image (i) shows haematoxylin and eosin staining of a tumour section; upper right image (ii) shows E2 staining which was predominantly cytoplasmic in the tumor and both nuclear and cytoplasmic in the stroma and infiltrating cells; lower left image (iii) shows the nuclear staining of ERα in the stromal cells only; lower right image (iv) shows aromatase expression detected in the cytoplasm of the tumour, stroma and infiltrating cells. Inset: normal rabbit serum negative control. Symbol T indicates tumour location in each picture; *Indicates stroma. Images are representative of n = 30.

The hormone oestradiol has been strongly implicated in the pathogenesis of human cervical cancer, but the exact role that E2 plays in tumor formation is currently unclear. In order to clarify how E2 promotes tumorigenesis in the human female genital tract, we first assessed levels of 17β-oestradiol in blood and tissue samples obtained from patients with squamous cell carcinoma (SCC) of the cervix. There was a significant difference in average concentrations of circulating hormone between patients and controls, however the levels were very low in both the groups (mean 26 pg/ml vs.39 pg/ml respectively; P < 0.002; (Fig. 1A.i). These data are consistent with previous reports that blood levels of oestrogen, although difficult to measure accurately at low concentrations, are known to be modulated in female cancers^13^. E2 concentrations in SCC tissue samples (mean 691 pg/100 mg, n = 30) were ~3 to 4-fold higher than those detected in tissue samples of normal cervix (172 pg/100 mg, n = 30;  P< 0.0001; Fig. 1A.ii) or healthy tissue sampled from sites adjacent to the tumours (240 pg/100 mg, n = 30; P < 0.0001) irrespective of patient’s age or menopausal status (13 of 30 study volunteers were post-menopausal women).

Having confirmed that E2 concentrations are increased in SCC tissues, we next investigated the cellular localization of the hormone using immunohistochemistry (IHC). For all cases of SCC tested (n = 30), IHC of tissue sections revealed marked E2 staining, which ranged in intensity from mild to moderate and was primarily located in the cytoplasm of tumour cells (30–80% stained E2 positive; (Fig. 1B.ii). Among tumour-infiltrating cell types, E2 staining varied from mild to strong and was more evenly distributed between the nucleus and cytoplasm. Nuclear E2 staining was detected in >80% of infiltrating inflammatory cells, which were comprised primarily of lymphocytes and fibroblasts (based on morphological criteria). Similar data were obtained when assessing tumour tissue sections for expression of the E2 biosynthetic enzyme aromatase, although enzyme staining was detected only in the cytoplasm (Fig. 1B.iv). Together, these data suggested that ongoing E2 synthesis in human cervical tumours may contribute to high local levels of this hormone despite the low concentrations present in blood plasma.

### Tumour-infiltrating T_reg_ cells express oestrogen receptor α and exhibit elevated levels of intracellular E2

**Figure 2:**
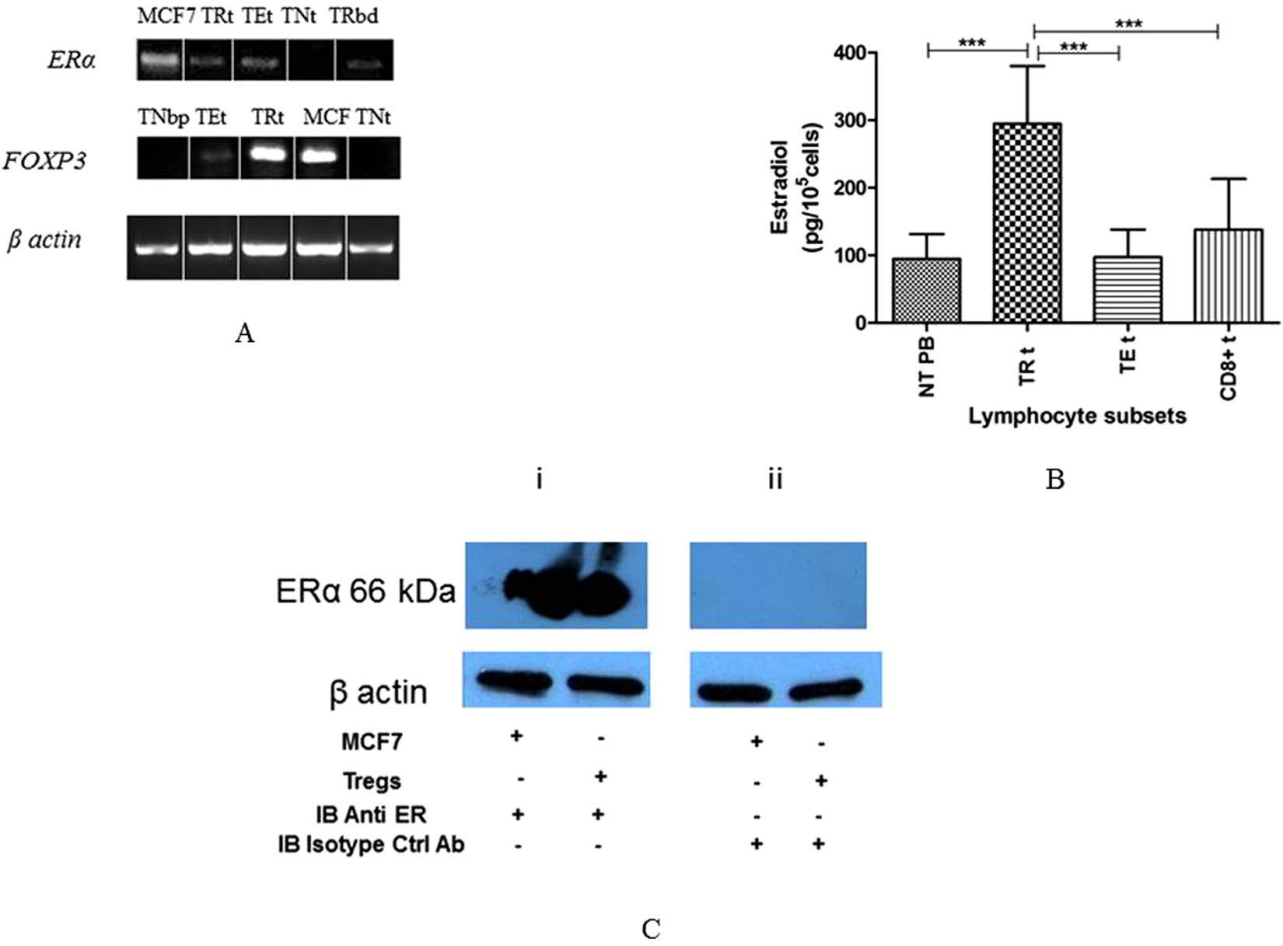
Primary human T_reg_ cells contain high levels of oestradiol hormone complexed with ERα. (**A**) Reverse-transcription PCR assessment of *ERα* and *FOXP3* expression in T_reg_ cells (TR), T_effs_ (TE) and Naïve T cells (TN) isolated from cervical tumours (t) or blood (b) obtained from CxCa patients (p) or healthy male donors (d). The *ERα*-expressing breast cancer cell line MCF7 was included as a positive control. Data are representative of 20 independent experiments. Gels have been run under the same experimental conditions. Full-length gels are presented in Supplementary Figs. S1 to S3. (**B**) E2 concentrations as quantified by ELISA in different T-cell subsets, MACS-purified and flow-sorted from tumour tissue of SCC cervix; T_reg_ cells - TRt (CD4 + CD25^hi^CD127^lo^), T_effs_ − TEt (CD4+CD25^int^), cytotoxic T lymphocytes (CD3+CD8+cells) and peripheral blood naïve T cells – NT PB (CD4+CD25^−^). A total of 10^5^ cells per subset were used for quantification. Graphs show mean ± SEM of n = 20 per subset (PB = peripheral blood; t = tumour). (**C**) Oestradiol complexes present in MACS/sort-purified tumour T_reg_ cells as detected by immunoprecipitation of oestradiol complexes using anti-E2 antibodies followed by immunoblotting with anti-ERα antibodies (i) or isotype-matched control antibodies (ii). β-actin immunoblots were carried out using lysates pre-immunoprecipitated with anti β-actin antibodies. Data are representative of 6 independent experiments. Gels have been run under the same experimental conditions. Please see Figs. S4 and S5 for original blot pictures.

Having confirmed E2 enrichment in cervical SCC tumours, we next assessed which of the infiltrating cell types expressed the corresponding oestrogen receptor - ERα and might be sensitive to modulation by E2 exposure. Sections of tumour tissue exhibited moderate ERα expression throughout the stroma and inflammatory cell infiltrate (30–50% staining in any given positive field), but staining was strongest in the nuclei of putative lymphocytes and fibroblasts which were distributed unevenly across the tumours (Fig. 1B.iii). In order to identify these ERα-expressing cell types, we next isolated the infiltrating lymphocytes from tumour tissues/peripheral blood by magnetic/flow-sorting and subjected these to reverse transcription PCR (primers listed in supplementary Table 1) to determine expression of *ERα* mRNA. Using this approach, we detected *ERα* expression in both CD8+ and CD4+ (CD4^+^CD25^int^) subsets of effector T-cells as well as in infiltrating T_reg_ cells (CD4^+^CD25^hi^CD127^lo^ cells) (Figs. 2A and S1a,b), suggesting that E2 sensitivity is prominent in antigen-experienced/regulatory cell types. Consistent with these data, we also detected marked *ERα* expression in peripheral blood T_reg_ cells whether obtained from female CxCa patients or healthy male volunteers (Figs. 2A and S1a,b).

We next assessed whether *ERα* expression by tumour-infiltrating lymphocytes was associated with hormone accumulation and potential E2 signalling in these populations. To do this, we isolated various T-cell subsets from CxCa tissues by magnetic/flow-based separation and quantified E2 levels in the cell lysates by competitive ELISA. We observed that mean concentration of E2 was ~2 to 3-fold higher in tumour-derived T_reg_ cells (294.4 pg/10^5^ cells) when compared with CD4+ effector T-cells (T_eff_ cells) (97.3 pg/10^5^ cells), CD8+ effector T-cells (138 pg/10^5^ cells), or peripheral blood naïve T cells (94.9 pg/10^5^ cells) (n = 20, P < 0.0001; Fig. 2B). These data indicated that human T_reg_ cells are enriched in intracellular E2, consistent with their high expression of ERα, suggesting that E2 hormone may have a significant role to play in the biology of regulatory T-cell subsets. Accordingly, we also detected high levels of E2 in T_reg_ cells separated from the peripheral blood of healthy male donors and in T_reg_ cells isolated from the draining lymph nodes (LNs) of patients with CxCa (data not shown). Together, these findings indicate that high concentrations of intracellular E2 are characteristic of human T_reg_ cell populations not only in CxCa tissues, but also in tumour-draining LNs and in the systemic circulation.

In addition, when we used anti-E2 antibodies to immunoprecipitate the hormone from cell lysates of CxCa-derived T_reg_ cells, subsequent immunoblotting with anti-ERα antibodies revealed a 66 kDa band consistent with receptor binding (Figs. 2C and S4 and S5). This finding confirmed that the E2 content of human CxCa-derived T_reg_ cells is at least partially complexed with the corresponding receptor ERα and may therefore exert an influence on the suppressive function of these cells.

### ERα regulates FOXP3 expression in human T_reg_ cells

**Figure 3:**
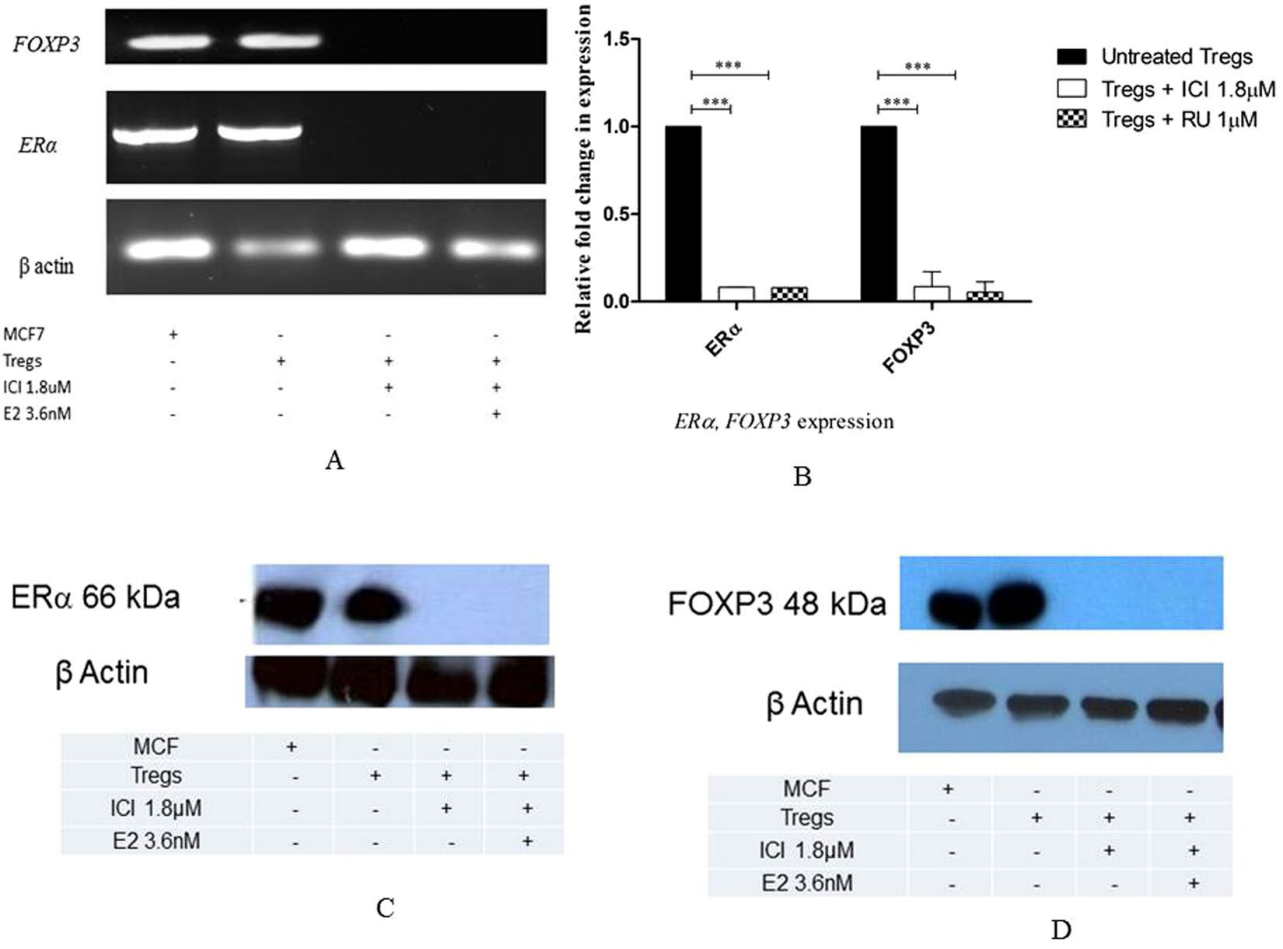
ERα control of FOXP3 expression in tumour-derived and peripheral blood T_reg_ cells. (**A**) *ERα* and *FOXP3* mRNA detection in CxCa T_reg_ cells or MCF7 cells after treatment or not with the ERα antagonist ICI 182,780 (ICI; 1.8 µM) for 72 h in the presence or absence of exogenous oestradiol (E2;3.6 nM). Data are representative of 6 independent experiments. Gels have been run under the same experimental conditions. Original gel images are shown in Figs. S6–S8. (**B**) Relative expression levels of *ERα* and *FOXP3* in peripheral blood T_reg_ cells subjected to the same treatment as described in Fig. 3A (n = 6 healthy male donors). (**C**) Loss of ERα protein expression in CxCa T_reg_ cells after 72 h treatment with ICI even in the presence of E2 supplementation throughout. Cellular extracts were immunoprecipitated using anti-ERα antibodies followed by immunoblotting with antibodies against ERα. The ERα-expressing breast cancer cell line MCF7 served as a positive control. β-actin immunoblots were carried out using lysates pre-immunoprecipitated with anti β-actin antibodies. Data are representative of 6 independent experiments. Gels have been run under the same experimental conditions. Please see Figs. S9 and S10 for original blot pictures (see also Fig. S11). (**D**) FOXP3 protein expression in CxCa T_reg_ cells after treatment or not with 1.8 µM ICI in the presence or absence of E2 for 72 h. Cell lysates were immunoprecipitated and immunoblotted using anti-FOXP3 antibodies. β-actin immunoblots were carried out using lysates pre-immunoprecipitated with anti β-actin antibodies. Data are representative of 6 independent experiments. Gels have been run under the same experimental conditions. Please see Figs.S12 and S13 for original blot pictures.

Pioneering experiments in mice have demonstrated that E2 can enhance the suppressive activity of T_reg_ cells by increasing *Foxp3* expression via a mechanism thought to depend on ERα^6,14^: however, *Esr1* knockout surprisingly did not result in complete disappearance of *Foxp3* ^6^. We therefore sought to determine whether E2 signalling through ERα is also capable of modulating *FOXP3* expression and suppressive function of human tumour-infiltrating T_reg_ cells in patients with CxCa. First, we analysed *FOXP3* mRNA in different T-cell subsets in the circulation and tumour mass, and we observed prominent *FOXP3* expression in T_reg_ cells in the peripheral blood and CxCa infiltrate, minimal expression in tumour-derived effector T-cells, and a complete lack of expression in naïve T-cell populations from either patients or donors (Figs. 2A and S2a,b). We therefore proceeded to test whether expression of *FOXP3* by T_reg_ cells could be decreased by exposure to the ER-specific pure antagonist ICI, which is already being used for therapeutic applications under the trade name Fulvestrant^15^. Using this approach, we observed that treatment with pharmacologically relevant concentrations of ICI^9,16^ abolished the expression of both *ERα* and *FOXP3* transcripts in both CxCa-derived T_reg_ cells (Figs. 3A and S6–S8), and in peripheral blood T_reg_ cells from healthy male volunteers (Fig. 3B). The loss of expression of both markers in male peripheral blood T_reg_ cells was also confirmed in experiments with another ER antagonist and Selective Estrogen Receptor Disruptor (SERD) - RU58,668 (Fig. 3B). We were also able to confirm the drug-induced loss of ERα protein in human T_reg_ cells by using anti-ERα antibodies to perform immunoprecipitation and immunoblotting assays (Figs. 3C and S9 and S10) as well as analysis by flow cytometry (Fig. S11). Consistent with the concept that E2:ERα signalling exerts a direct influence on human T_reg_ cell function, we also observed that ICI treatment ablated FOXP3 protein expression as revealed by immunoprecipitation and immunoblotting using anti-FOXP3 antibodies (Figs. 3D and S12 and S13). Furthermore, supplementing these T_reg_ cells with physiological concentrations of E2 was unable to rescue gene expression of either *ERα* or *FOXP3* (Figs. 3A–D; S6 to S13). Together, these results indicated that ERα critically regulates the expression of FOXP3 in human T_reg_ cells.

### Intracellular oestradiol-liganded oestrogen receptor regulates CxCa T_reg_ cell function

**Figure 4:**
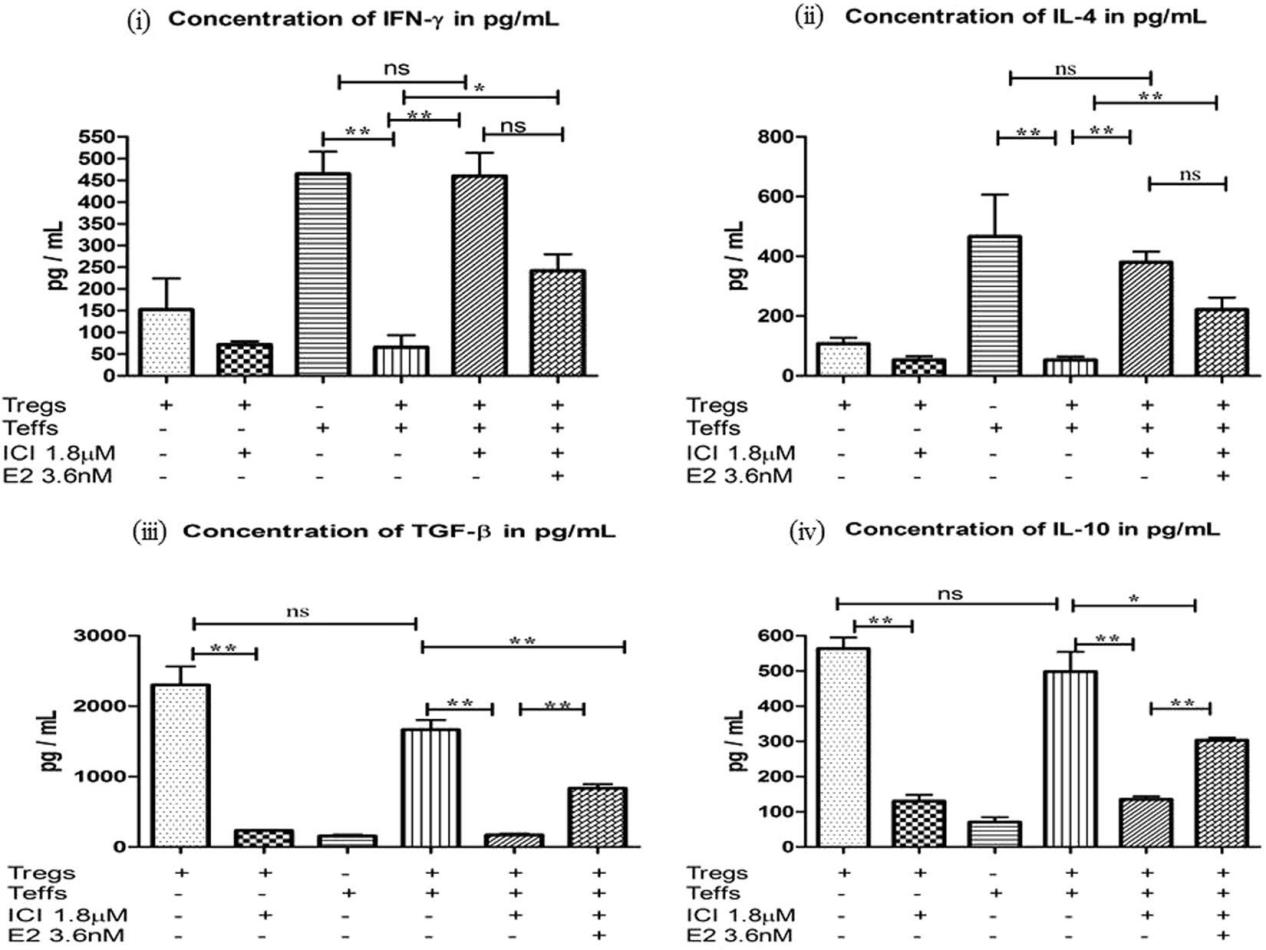
ERα and E2 modulate human T_reg_ cell function. Equal numbers of tumour-dervied T_reg_ and T_eff_ cells (1 × 10^5^ cells per subset) were co-cultured with or without ICI and exogenous E2 for 5 days and cytokine concentrations in the supernatant were determined by ELISA. Graphs show mean ± SEM of n = 6 experiments (see also Fig. S14).

**Figure 5:**
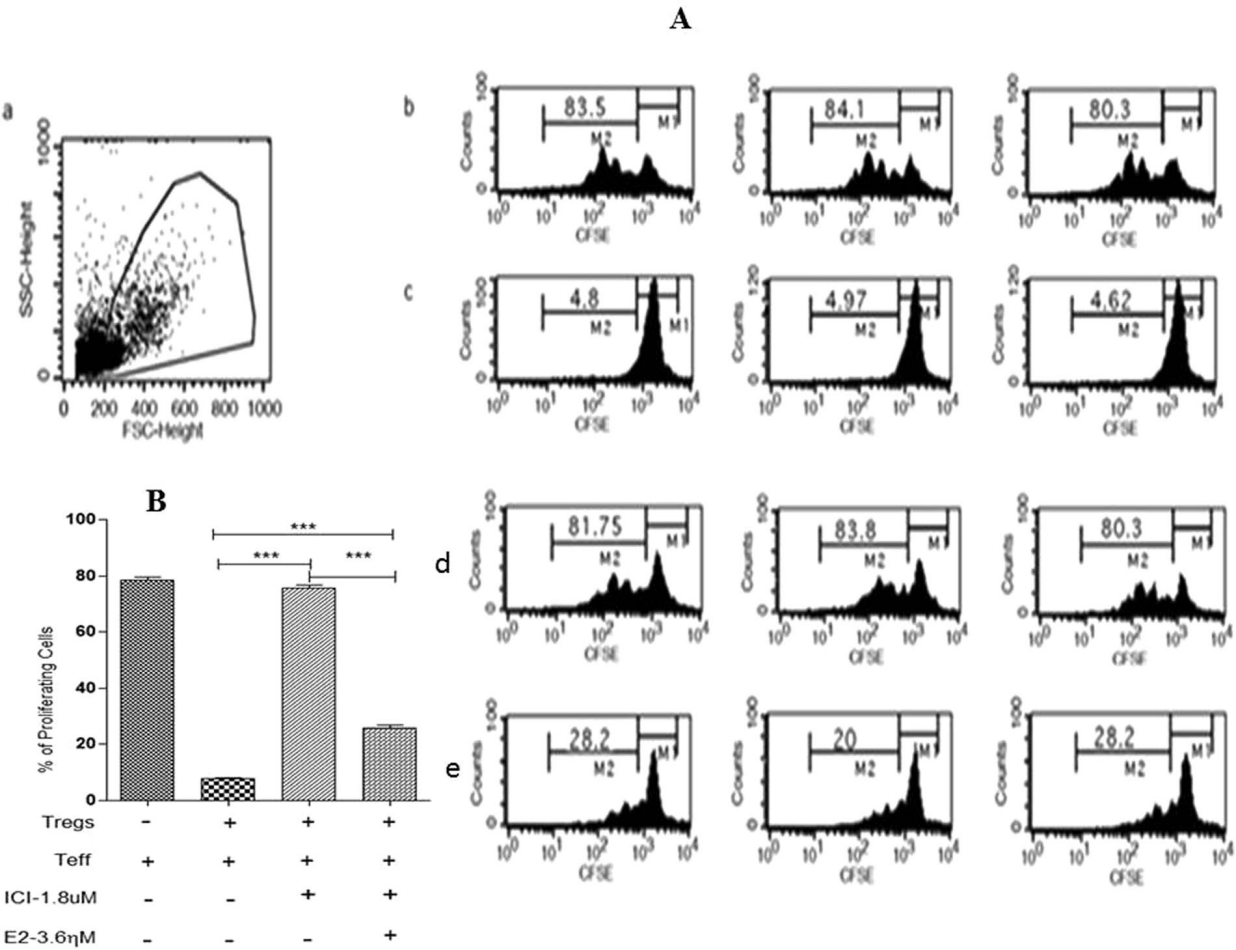
ERα and E2 modulate human T_reg_ cell proliferation. (**A**) Effect of ICI and E2 treatment on T_reg_ cell’s suppressive function during 5 days co-culture with CFSE-labelled T_eff_ cells. a: Lymphocyte gating based on light scatter; b: Percentage of T_eff_ cells proliferating in response to stimulation with anti-human CD3/CD28. c: T_reg_ cell inhibition of T_eff_ cell proliferation during co-culture (1:1). d: T_eff_ cell proliferation in co-culture with ICI-treated T_reg_ cells (1:1). e: rescue of T_reg_ cells’ suppressive function in co-cultures with T_eff_ cells (1:1) treated with 1.8 µM ICI and 3.6 nM E2. M1: Percentage of non-proliferating T-cells; M2: Percentage of proliferating T-cells. Shown is a representative example of the FACS analysis (See also Figs. 5B and S15 and S16). (**B**) Percentage proliferating T_eff_ cells in co-culture with T_reg_ cells after treatment or not with ICI and exogenous E2. Mean ± error bars of 6 independent experiments performed in triplicate. (See also Figs. S15 and S16).

We have previously demonstrated that in co-cultures of CxCa-derived T_eff_ and T_reg_ cells, effector cell secretion of IL4 and IFNγ is efficiently suppressed by regulatory cells that produce TGFβ and IL10^17^. We therefore sought to investigate whether E2:ERα signalling effects on T_reg_ cell function might contribute to the restriction of anti-tumour effector T-cell responses in human CxCa. In co-cultures of CxCa-derived T_reg_ and T_eff_ cells, we observed that ICI induced a significant drop in the expression of suppressive cytokines TGFβ and IL10 (~10 and 4-fold decrease respectively, P < 0.01), and increased production of effector T-cell cytokines IL4 and IFNγ (~7-fold rise in both cytokines, P < 0.01) perhaps indicating restoration of effector potential upon inhibition of tumour-derived T_reg_ cells (Fig. 4). We also observed a dose-dependent decrease in TGFβ production by CxCa-derived T_reg_ cells when exposed to ICI either in the presence or absence of autologous T_eff_ cells (Fig. S14). The significant reduction in TGFβ output in the presence of ICI was accompanied by a progressive decrease in the suppressive function of CxCa T_reg_ cells, which displayed a dose-dependent decline in their ability to restrict the proliferation of autologous T_eff_ cells (P < 0.001; Figs. 5A,B and S15). Annexin V staining demonstrated that ICI-treated T_reg_ cells maintained high levels of cell viability throughout culture (~95% at 1.8 µM ICI; Supplementary Fig. S16), hence the loss of cytokine secretion and suppressive potential of CxCa-derived T_reg_ cells could not be attributed to cell death. Indeed, E2 supplementation in co-cultures of T_eff_ cells and ICI-treated T_reg_ cells partially restored secretion of suppressive cytokines (P < 0.01) and restriction of CD4+ effector T-cell proliferation (P < 0.001; Figs. 4, 5A,B and S15; P < 0.001).

Together these data indicated that E2:ERα signalling effects on *FOXP3* expression in human CxCa T_reg_ cells alter cytokine secretion by these cells to modulate their regulatory function. However, since we have previously established that only ~50% of CxCa-infiltrating T_reg_ cells (CD4+CD25^hi^CD127^lo^ cells) express *FOXP3* as measured by flow-cytometry^17^, we cannot exclude additional effects of the hormone on FOXP3-negative T_reg_ cells or rule out the possibility that E2 may also act via non-ERα signalling pathways in CxCa T_reg_ cells. Nonetheless, these data provide compelling evidence that intracellular complexes of E2:ERα play a vital role in the functional regulation of human FOXP3+ T_reg_ cells that infiltrate human CxCa tumours.

### Oestrogen receptor alpha binds the *FOXP3* locus and expressed protein

Since oestrogen receptor is known to modulate the expression of multiple genes by binding directly to target promoters, we next sought to clarify whether ERα effects on *FOXP3* expression in T_reg_ cells were mediated by binding to the corresponding promoter. To do this, we used chromatin immunoprecipitation-coupled quantitative PCR (ChIP-qPCR) to map potential ERα binding sites at the *FOXP3* promoter of primary human T_reg_ cells. Since only limited numbers of T_reg_ cells can be isolated from CxCa tissues, and these cells are not uniformly FOXP3+^17^, we performed these analyses using CD4+CD25^hi^CD127^lo^ T_reg_ cells separated from the peripheral blood of healthy male donors.

**Figure 6:**
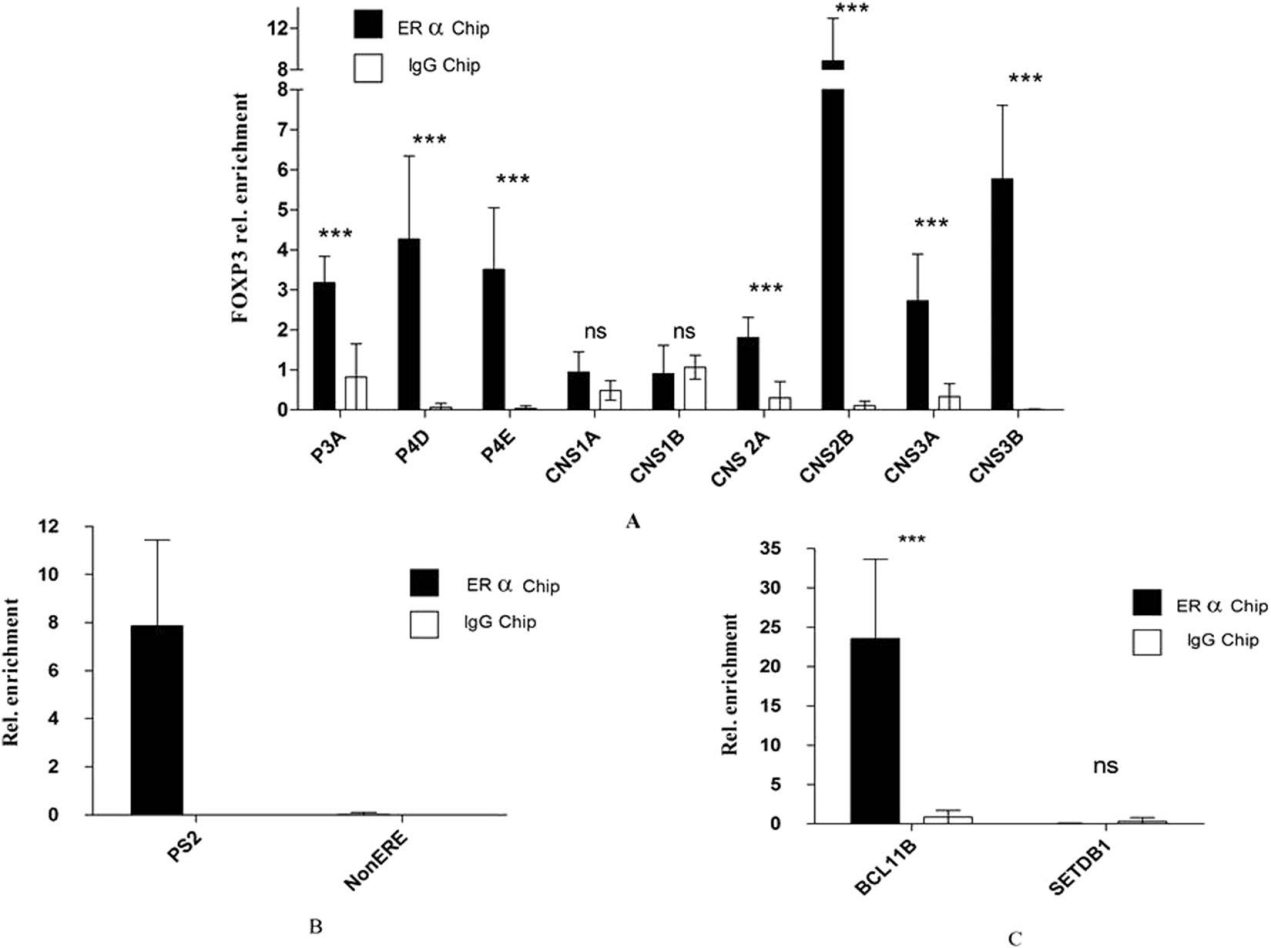
ERα-enriched regions of the *FOXP3* locus. ChIP-qPCR analysis of the *FOXP3* promoter and enhancer regions in chromatin fragments pulled down by anti-ERα antibodies (filled bars) or control IgG (open bars) in the cell lysates of blood T_reg_ cells obtained from healthy male volunteers (**A** and **C**) or MCF7 cells (**B**). Data shown are mean ± SEM of 6 independent experiments performed in triplicate.

Using primer pairs targeting different regions of the *FOXP3* promoter and enhancer regions (Supplementary Table 2), we identified ERα binding at multiple sites along the locus, including regions both upstream and within the core promoter, as well conserved non-coding DNA sequence elements (CNS)-2 and CNS3, but not CNS1 (Fig. 6A). These data likely reflected genuine ERα interaction with the *FOXP3* locus, since we also detected receptor binding to known oestrogen response elements (EREs) at the loci *BCL11B* and *pS2* in primary human T_reg_ and MCF7 cells, but not to the *SETB1* and *FKBP6* genes respectively which lack the ERα target sequence (Fig. 6B and C). Together, these data indicated that ERα interaction with the *FOXP3* locus occurs primarily at sites both upstream and within the core promoter, as well as at the regions CNS2 and CNS3.

When we searched for ER-responsive elements of the *FOXP3* promoter/enhancer using *in-silico* analysis of the enriched regions, we detected eight putative ERα binding sites distributed between regions upstream of the core promoter, adjacent to the TSS, and at CNS2 and CNS3 (P < 0.01)^18^. The majority of these putative ERα binding sites exhibited sequences characteristic of ‘ERE half-sites’, which have been reported to exhibit strong affinity for ERα binding^19^. Many of these EREs were also found to be conserved between the human, mouse and rat genomes (Figs. S17–S20; Supplementary Table 3). The enriched region upstream of the core promoter incorporated two ERE half-sites at positions −943 and −846 (Fig. S17), whereas the core promoter itself included conserved binding motifs at +39 and +114 bp downstream to the TSS (Fig. S18). CNS2b harboured a conserved ERE half-site at +4030 and was strongly enriched in ERα binding relative to CNS2a, which displayed a full ERE at +3895, suggesting that the former region may be critical for receptor binding to the *FOXP3* locus (Fig. 6A and S19). Given the marked ERα enrichment at CNS3b (Fig. 6A and S20) and evolutionary conservation of both putative ERE half-sites, it seems likely that one or both of these sites is crucial for ERα binding to CNS3.

**Figure 7:**
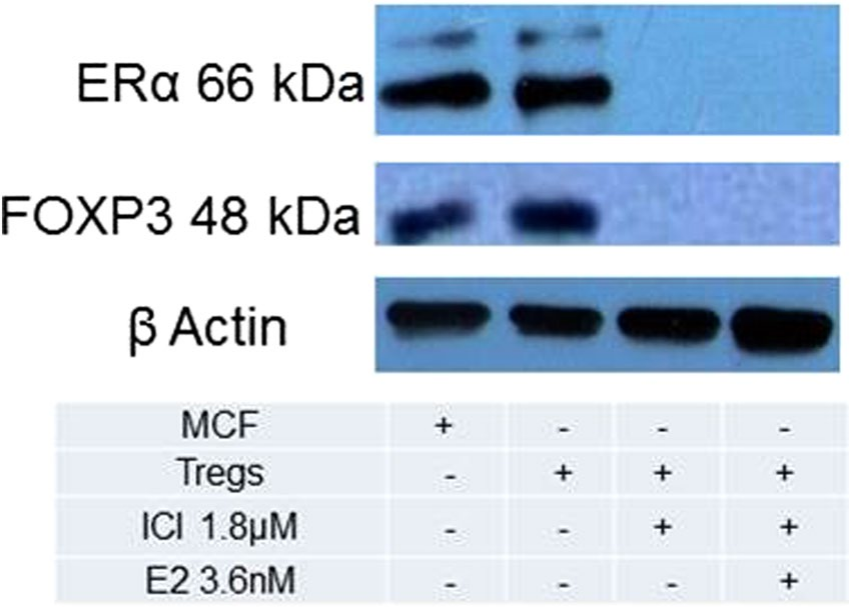
ERα protein interacts with FOXP3 protein in human tumour-derived T_reg_ cells. CxCa T_reg_ cells were treated or not with ICI in the presence or absence of exogenous E2 for 72 h before lysis. The cell lysates were then immunoprecipitated using anti-E2 antibodies and the protein complexes obtained were immunoblotted using antibodies against ERα and FOXP3. β-actin immunoblots were carried out using lysates pre-immunoprecipitated with anti β-actin antibodies. Data are representative of 6 independent experiments. Gels have been run under the same experimental conditions. Please see Figs. S21 to S23 for original blot pictures. See also Fig. S24.

Since FOXP3 is known to auto-regulate its own promoter by binding to CNS2 region, we next sought to probe the physical interaction of E2 hormone and ERα with FOXP3 in primary human T_reg_ cells isolated from CxCa tissues. To do this, we conducted co-IP experiments using the total cell lysates of T_reg_ cells magnetically/flow sorted from CxCa tissues. We detected both ERα and FOXP3 in the immunoprecipitates obtained using anti-E2 antibodies (Figs. 7 and S21 to S23). Similarly FOXP3 was successfully detected in the anti-ERα immunoprecipitates (Fig. S24), thus indicating that ERα exists in a form complexed with FOXP3 in CxCa-derived T_reg_ cells. Importantly, antibodies against E2 or ERα were unable to immunoprecipitate FOXP3 protein from the cell lysates of T_reg_ cells that had been treated with ICI (Figs. 7 and S21 to S24). Together, these *in silico* and experimental data suggest that ERα may form part of an auto-regulatory loop in which the interaction of FOXP3 protein and E2:ERα complexes with the *FOXP3* locus contribute to the maintenance of transcription factor expression^20^, and potentially influence the clinical course of human cervical cancers.

## Discussion

In the current report, we provide evidence that E2:ERα complexes drive *FOXP3* expression and suppressive function of primary human T_reg_ cells in both healthy male individuals and in tumours from patients with CxCa. Our data suggest that ERα critically regulates *FOXP3* expression by binding to multiple elements along the locus as well as by directly interacting with FOXP3 protein intracellularly. Collectively, these findings uncover a pivotal role for ERα in T_reg_ cell biology that may have important implications for the therapeutic targeting of these cells in patients with various types of cancer including CxCa.

It has been widely reported that various solid tumours exhibit oestradiol accumulation despite normal levels of this hormone being present in the circulation^21^. Oestradiol up-regulation in tumours is due to increased uptake and retention of circulating oestrogens E1 and/or E2, as well as enhanced hormone biosynthesis via the action of aromatase enzyme locally in the tumours^22^. Indeed, we detected marked aromatase expression in the tumour tissues analysed here, consistent with an earlier report that enzyme levels are increased in human CxCa^23^. Accordingly, the hormone products of the aromatase pathway have been strongly implicated in the pathogenesis of CxCa in both an animal model and in human patients^24–26^. In-line with these earlier findings, we detected high levels of E2 in human CxCa tumour tissue, whereas hormone levels in blood plasma remained low in both cancer patients and healthy volunteers. More surprising was our novel finding that primary human T_reg_ cells are also characterized by high levels of intracellular E2, both in the peripheral circulation and CxCa tumours, suggesting that this hormone may have an important role to play in suppression of host anti-tumour immunity.

The cellular response to E2 hormone is predominantly mediated by the intracellular receptors ERα and ERβ, as well as the membrane G protein-coupled Oestrogen Receptor. In-line with the findings of a previous report^27^, our immunohistochemical analyses of CxCa tissues confirmed that both E2 hormone and ERα were primarily localized in the nuclei of stromal fibroblasts and tumour-infiltrating lymphocytes (TILs), and analysis of purified cell subsets identified strong expression of E2 and ERα in isolated T_reg_ cells. In partial agreement with these data, a recent tissue microarray analysis of human CxCa tumours identified ERα expression by stromal fibroblasts, but failed to detect receptor expression by CD45+ hematopoietic cells^28^, perhaps due to the non-uniform distribution of FOXP3+cells within the tumours^29^. Previous studies in mice and human blood have shown that T_reg_ cells express both ERα and ERβ, but population expansion triggered by E2 exposure is primarily mediated via ERα signalling^6,8,30^. Accordingly, when we used a novel approach of using anti-E2 antibodies to capture proteins that were complexed with this hormone in blood and tumour-derived T_reg_ cells, we observed that intracellular E2 was at least partially complexed with ERα, and might therefore be capable of modulating T_reg_ cells function via this pathway.

The hypothesis that E2 could induce *FOXP3* expression was first proposed over a decade ago^6^. Direct evidence of this mechanism and its relevance in humans have until now remained obscure particularly since studies in *Esr1* knockout mice did not abolish *FOXP3* expression^6^. In the current report, we demonstrated that treatment with a SERD was able to abolish *FOXP3* expression in T_reg_ cells isolated from tumour tissues or peripheral blood obtained from CxCa patients or healthy volunteers, suggesting a direct influence of the classical ERα signalling pathway on sustenance of *FOXP3* expression and T_reg_ cell function. This is in contrast to studies in mice wherein basal expression of *FOXP3* was retained in *Esr1* knockout animals^6^. We could argue that this baseline expression could have had its origin in cord blood T_reg_ cells where *FOXP3* expression is progesterone dependent^31^. Hence these experiments unambiguously indicated that ER signaling through the classical pathway controlled *FOXP3* expression in T_reg_ cells. Loss of *FOXP3* expression has previously been shown to result in a corresponding loss/dysfunction of T_reg_ cells in mice and humans respectively^32–34^. E2:ERα signalling effects on *FOXP3* expression are therefore likely to exert a significant influence on T_reg_ cells function in human CxCa. However, therapeutic targeting of this pathway is likely to be complicated by reports from our lab and others that the T_reg_ cell population of solid tumours can include both FOXP3+ and FOXP3^−^ cells^17,35^. Accordingly, we observed that supplementation with E2 induced only a partial revival of suppressive function in ICI-treated T_reg_ cells, suggesting additional effects of the hormone that do not depend on ERα and/or FOXP3^11,12^. Our data suggest that ERα ranks among a select group of cellular factors, including TGF-β and NR4A2 that can exert major effects on T_reg_ cell’s expression of *FOXP3* ^36^. Despite the known ability of TGFβ to induce *Foxp3* expression in pT_reg_ cells, this cytokine is unable to increase *Foxp3* levels in tT_reg_ cells^37^, and failed to sustain *FOXP3* expression by CxCa-derived T_reg_ cells upon ERα inhibition in the current study. These data suggest that FOXP3+ T_reg_ cells residing in CxCa tissues are tT_reg_ cells rather than pT_reg_ cells^3^, which may have implications for the therapeutic manipulation of tumour T_reg_ cell function in human patients.

Our ChIP-qPCR analyses of the *FOXP3* locus revealed ERα occupancy of four distinct regions; upstream of the core promoter, within the core promoter, and at CNS2 and CNS3. By combining data from the literature with TRANSFAC and/or Jasper analysis, we identified Oestrogen Responsive Elements that not only appeared to be conserved across the mammalian species, but were also located in the vicinity of other known transcription factor binding sites proposed to influence ERα binding, *FOXP3* expression, and/or T_reg_ cell function^20,38–43^. In particular, the CNS2 region features binding sites for numerous transcription factors known to interact with ERα^44,45^, and may form short distance loops to interact with the core promoter and induce *FOXP3* gene expression^46,47^. Less clear are the roles of the putative binding sites at CNS3 and in regions upstream of the core promoter, although future studies should be able to determine whether these are involved in stabilizing DNA looping and possibly facilitate interactions between other critical regions. Global analyses of receptor occupancy and expression profiling may also uncover additional gene targets of ERα modulation in human T_reg_ cells with potential for novel clinical applications.

**Figure 8:**
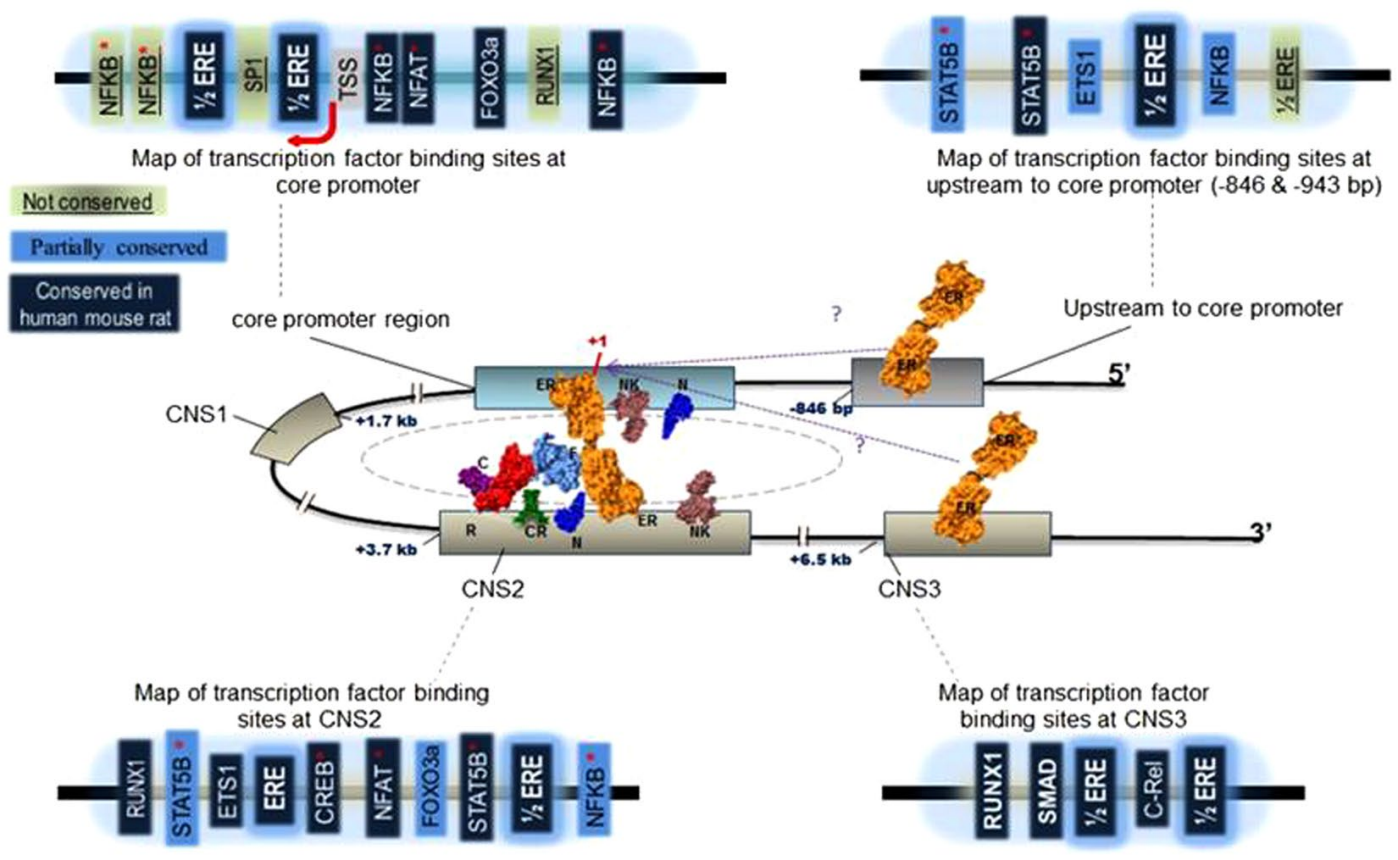
Diagrammatic representation of proposed ERα interactions with the *FOXP3* locus in human T_reg_ cells: RUNX1(R), CBFβ (C), Oestrogen Receptor (ER), CREB (CR), NFAT (N), NFKB (NK) and FOXP3 (F). Transcription factors that have previously been suggested to be involved in the looping of relatively distant DNA elements near the core promoter are marked with ‘*’. See also Figs. S17 to S20.

Earlier studies have demonstrated that complexes of Foxp3 protein with Runx1 and Cbf-β bind to the de-methylated CNS2 region of the *FOXP3* locus to ‘auto-induce’ gene expression^20,48^, and proteomic studies have identified at least 361 interacting partners of Foxp3 in T_reg_ cells^49^. In our study, we observed that in addition to ERα binding to the *FOXP3* locus in human T_reg_ cells, E2:ERα complexes also physically interacted with FOXP3 protein in these cells. Other known co-activators of ERα include the histone acetyl transferases (HATs) p300/CBP^50^, which act on the CNS2 region of the *Foxp3* locus to maintain T_reg_ cell stability^51^. We are therefore tempted to speculate that the *FOXP3* locus in T_reg_ cells is maintained in an active state by HAT-mediated recruitment of ERα to FOXP3-RUNX1-CBFβ complexes at CNS2. In light of these data, as well as earlier reports, we propose a model in which multiple ERα binding sites flanking the *FOXP3* promoter regulate gene activity through a combination of direct DNA binding and indirect tethering/looping in a manner similar to that reported for ER-regulated genes in MCF-7 breast cancer cells (Fig. 8)^44,52^.

Cervical cancer remains a major burden globally especially in developing countries. Currently, chemo-radiation is the mainstay of treatment; however, five year survival rates are low as most of the patients present with advanced disease. Immunotherapy is a useful alternative to treat the disease. However, simultaneous to potentiation of effector responses in the tumor microenvironment, cancer immunotherapies must also overcome host immune suppression mediated by T_regs_ cells. Accordingly, we have presented evidence that E2:ERα interactions with *FOXP3* locus exert potent effects on gene expression and can modulate the suppressive function of primary human tumor infiltrating T_reg_ cells in patients with CxCa. These data not only advance our current understanding of basic T_reg_ cell biology, but will also inform attempts to target ERα for therapeutic benefit in CxCa as well as other solid tumours (unpublished data from our lab has revealed elevated concentrations of the hormone E2 in various types of solid tumors in males), autoimmune diseases, and inflammatory disorders in which T_reg_ cells play a central role. Also, in support of this proposed line of action are recent studies including ours showing paracrine ERα signaling in Cancer Associated Fibroblasts (CAFs) to be relevant in human CxCa^28,53^. Hence, we envisage that it may be worth exploring targeted therapy with ER antagonist in the management of CxCa: interfering with both T_reg_ cell function and paracrine signaling through fibroblast ERα may help in checking the growth of cancer cells. Although we did not assess the effect of ICI on tumor T_eff_ function which also expressed *ERα*, we envisage that ER antagonists would have an additive effect of boosting tumor infiltrating Th1 subset as well^54^. We therefore believe that the potentially far-reaching implications of our study *have great translational value*.

## Materials and Methods

### Study Design

#### Subjects

Fresh cervical tumour tissue (FIGO stage IB2), draining lymph nodes, and whole blood were obtained from patients undergoing radical hysterectomy for invasive cervical cancer. Normal cervices were obtained from women undergoing hysterectomy for benign conditions. Additional whole blood samples were collected from healthy male donors and used for isolation of T_reg_ cells. Written informed consent was obtained from all study participants. The study was approved by Institutional Medical Ethics Committee (Kidwai Memorial Institute of Oncology, Bangalore, India). All experiments were performed in accordance with relevant guidelines and regulations.

*Replicates:* All experiments were performed in triplicates.

### Oestradiol quantification

Oestradiol was extracted and quantified according to the procedure described by Rao *et al*.^55^. Briefly, 100 mg tumour tissue or 10^5^ sorted cells were homogenized in 1 ml digestion buffer (0.1 M Phosphate Buffered Saline [PBS], pH 7.4, containing 100 mM EDTA, 100 μg/ml proteinase K [No. P2308, Sigma, St. Louis, MO], and 250 μg/ml DNase [No. DN5025, Sigma]) for 30 mins at 37^o^C before being subjected to chemical-based Oestradiol extraction. A total of 3 ml di-ethyl ether was added to the lysate and the mixture was snap-frozen in liquid nitrogen for 10 min. Upon thawing, the supernatant was collected and left at room temperature overnight to allow ether evaporation. The hormone-containing pellet was re-suspended in 1 ml PBS containing 10% gelatin and then used for Oestradiol quantification using Chemiluminescence Immuno Assays (no. 03000079190, Elecsys Oestradiol II, COBAS, USA) or Oestradiol competitive ELISA kits (no: 74070, Equipar, Saronno VA; Italy).

### Immunohistochemical (IHC) staining of Oestradiol, ERα, and Aromatase

IHC staining of tissue sections of human cervical SCC was performed using the following antibodies (Abs): rabbit anti-human oestradiol (no. AR038-5R) and rabbit anti-human oestrogen receptor α (no. AM272-2M) from Biogenex, CA, USA; and rabbit anti-human aromatase (no. Ab35604), from Abcam UK. Secondary Abs (no. BA-1000) and ABC-Peroxidase kit VECTASTAIN® (no. PK-4001) were from Vector Laboratories Inc., CA, USA. Immunohistochemistry was performed as previously described^17^. Images of the stained sections were captured using an Olympus Bx 500 microscope and staining intensity/distribution in the tumour and stroma were scored by a histopathologist using Image Pro Plus software (Media Cybernatics, Rockville, MD, USA).

### Cell Isolation, Purification and Analysis

Tumour-infiltrating lymphocytes (TILs) were isolated from enzymatically digested tumour tissues as detailed in our earlier report^17^. Additional procedures adopted for the isolation of specific cell types are described below:

### T_reg_ cells (CD4+CD25^hi^ CD127^lo^ cells)

Cell suspensions prepared from homogenized tumour tissues or peripheral blood samples were initially enriched for T cells by passing through nylon wool columns. Putative T_reg_ cells were then isolated from the total T cell pool using a MACS CD4+CD25+ Regulatory T Cell Isolation Kit (no. 130-093-337, Miltenyi Biotec, GmBH, Germany). MACS-enriched CD4+CD25^hi^CD127^lo^ T_reg_ cells isolated from peripheral blood were used without further purification, whereas tumour-derived T_reg_ cells were additionally subjected to flow sorting to obtain high purity CD4+CD25^hi^ cells. We have previously determined the purity of MACS-enriched CD4+CD25^hi^CD127^lo^ cervical tumour T_reg_ cells to be 96% pure as assessed by flow cytometry^17^.

### T_eff_ cells (CD4+CD25^int^)

Nylon wool-enriched TILs were flow-sorted to obtain CD4+CD25^int^ cells without prior MACS enrichment. Uniform sort gates were applied to all samples and cell purity was confirmed by re-acquisition of the sorted populations^17^.

### Naïve T-cells (CD4+CD25^−^), B cells and CD8+ T-cells

CD4+CD25^−^ naïve T cells, B cells, and CD8+ T cells were isolated from blood samples and/or CxCa tumour tissues using MACS kits (Miltenyi nos. 130-094-131, 130-091-151 and 130-096-495 respectively) as per the manufacturer’s instructions. Naïve T cells were additionally flow-sorted to obtain a highly pure CD4+CD25^−^ population.

### Flow cytometry

Lymphocyte subset analysis was performed using a FACS calibur^TM^ flow-cytometer (Becton Dickinson, San Jose, USA). Cell sorting was conducted using a FACS Vantage SE apparatus (Beckton Dickinson, San Jose, USA.).

### T_reg_ cell suppression assays

CD4+CD25^int^ T_eff_ cells isolated from CxCa tissues were used as responder cells in T_reg_ cells suppression assays. T_eff_ (5 × 10^4^) cells were cultured with or without an equal number of T_reg_ cells and 5 × 10^3^ autologous B cells to serve as antigen presenting cells. In some experiments, T_reg_ cells were incubated with different concentrations of ICI 182,780 (36.7 nM, 367 nM, 1.8μM; No.I4409, Sigma) in the absence or presence of E2 hormone (3.6 nM; kind gift from Prof. A J Rao) for 30 min at 37 °C, 5%CO_2_ before being washed and co-cultured with T_eff_ that had been pre-labelled with CFSE (1 μg/ml; 21888, Sigma). The cultures were then incubated for 5 days in indicator-free RPMI-1640 medium (No. R8755, Sigma), containing 10% charcoal-stripped Foetal Calf Serum (FCS; F2442, Sigma), and 300U/ml recombinant human IL2, and stimulated with plate bound anti-CD3ε (1 μg/ml functional grade purified clone OKT3) and anti-CD28 (0.5 μg/ml functional grade purified clone CD28.2, eBiosciences Inc. San Diego, CA, USA). Responder cell proliferation was assessed by CFSE dilution measured using a MoFlo flow cytometer (DAKO, USA). Cell divisions were quantitated using summit V4 software (DAKO cytomation, USA) and the results expressed as mean percentage proliferating cells ± SEM of triplicate cultures. RU 58,668 (1 μM; 3224, Tocris Bioscience) was used to confirm the results obtained with ICI^56^. Stock solutions of ICI, RU and oestradiol were prepared in ethanol and serially diluted in culture media to obtain the specified concentrations.

### Cytokine quantitation in cell culture supernatants

After 5 days incubation, the culture supernatants were aspirated and assayed for concentrations of the cytokines IFNγ, IL4 and IL10, TGFβ1 (latent form) using the following ELISA kits; Human IFNγ (no. 88–731); Human IL4 (no. 88–7046); Human IL10 (no. 88–7106); and Human LAP (TGF β1) (no. 88–50390), (all from eBiosciences), as per the manufacturer’s instructions. Mean values of triplicate measurements were tabulated.

### PCR determination of *FOXP3* and *ERα* mRNA expression

Total cellular RNA was extracted using TRIzol reagent (no.15596-026, Ambion, New York, USA). Reverse transcription PCR determination of *FOXP3* and *ERα* mRNA levels was performed using oligo dT and Moloney Murine Leukaemia Virus reverse transcriptase (no. 210210; Qiagen). All samples were run in triplicate. MCF-7 cell line which expresses *ER* *α* and Human FOXP3 plasmids pCMV-Tag2-human FoxP3, which generates full-length human FoxP3 by EcoRI and SalI, and retroviral pMCsIg (IRES-GFP)-human FoxP3 (kind donation from Dr. Ono M) were used as positive controls. Expression of *β actin* gene served as internal control. Primer sequences are provided in Supplementary Table 1 ^57–59^.

### Real-Time quantitative Reverse Transcription PCR

Messenger RNA levels of *FOXP3* and *ERα* were determined by real-time PCR using a Step One Plus thermal cycler (Applied Biosystems). Briefly, 250 ng of total RNA was reverse transcribed, 10-fold diluted in sterile nuclease free water, and 2 μl used per 10 μl reaction volume with 1X Power SYBR Master Mix, and 100 nM of forward and reverse primers (listed in Supplementary Table 1). Cycling conditions were: 1 min at 95 °C, followed by 40 cycles of 15 s at 95 °C denaturation, 60 s at 60 °C annealing. Samples were run in duplicate and expression levels were determined using the 2^ΔΔCt^ method with *GAPDH* as the internal control^60,61^.

### Co-immunoprecipitation assays

Capture antibodies (*directed against epitopes at/near the N termini of the respective target proteins*); Rabbit anti-E2 (AR038-5R, BioGenex); mouse anti-human FOXP3 (clone 259D/C7, BD Pharmingen, NJ, USA); and rabbit anti-human ERα clone 1D5 from Biogenex (NU509-UCE) or Dako Cytomation, CA, USA (M7047).

Probing antibodies (*directed against epitopes at/near the C termini of the respective target proteins*); mouse anti-human ERα (clone F10, Santa Cruz Biotechnology Inc, USA); and rabbit anti-human FOXP3 (ab10563; Abcam, against epitope aa 400).

Detection antibodies: goat anti-mouse IgG HRP and goat anti-rabbit IgG HRP (31436 and 31466 respectively, from Pierce Biotechnology Inc., USA). TrueBlot® anti-rabbit and anti-mouse Ig IP Beads (nos. 008800 and 00881125 respectively) were from eBiosciences.

For immunoprecipitation assays, total cellular protein extracts were first obtained using various methods. To assess whether E2 was complexed to ERα, primary tumour-derived T_reg_ cells were purified by MACS/FACS and then lysed. To assess the extent of ERα co-localization with FOXP3, tumour-derived T_reg_ cells were incubated in the presence of different concentrations of ICI and/or E2 hormone (or ethanol-only control) prior to stimulation for 72 h with plate bound anti-CD3 (1 μg/ml), anti-CD28 (0.5 μg/ml) and recombinant human IL2 (100 IU/ml) prior to lysis. The protein extracts were then processed for immunoprecipitation using antibody-coated IP beads according to the manufacturer’s instructions. Immunoprecipitated material/protein extracts were separated on 10% SDS-PAGE gels and transferred onto Biodyne C nylon membranes (no. 60315, Pall Life Sciences, New York, USA). The membranes were probed with antibodies and visualized using the super signal^®^ west pico chemiluminescent substrate (no. 34079, Thermo Scientific, Rockford, USA). The MCF-7 breast cancer cell line was used as a positive control both for FOXP3 and ERα detection. SDS sample buffer was used as the negative control.

### Cell viability staining

Propidium iodide (PI) and annexin V–FITC apoptosis detection kits (no. K101; BioVision Inc, California, USA) were used to determine the viability of ICI treated T_reg_ cells isolated by MACS/FACS sorting from tumour tissues. Cell staining was determined by flow cytometry.

### ERα staining using flow cytometry

For assessment of ERα expression by flow cytometry, a total of 2 × 10^5^ T_reg_ cells were cultured for 72 h with various concentrations of ICI *182, 780* (36.7 nM, 367 nM, or 1.8μM), then surface staining with anti-human CD4-PE (clone OKT4, eBiosciences), followed by permeabilization and intracellular staining with rabbit anti-human ERα antibodies (no. ab37438, Abcam). After 30 min incubation, the cells were stained for a further 30 min with goat anti-rabbit IgG-FITC conjugated antibodies (No. F6005, Sigma) and washed in 1x PBS containing 0.1% BSA to remove excess/unbound antibodies. A total of 20,000 cells were acquired using a BD FACS Calibur apparatus and lymphocyte staining for ERα was determined relative to lymphocytes treated with normal rabbit/goat sera.

### Chromatin Immunoprecipitation (ChIP)

CD4+CD25^hi^CD127^lo^ T_reg_ cells were isolated from the peripheral blood buffy coats of healthy donors using the MACS T_reg_ cell isolation kit (no. 130-093-337, Miltenyi). The ERα-expressing breast cancer cell line MCF7 was used as a positive control. Chromatin was sheared in a Bioruptor (Diagenode, Belgium) using 12 pulses of 30 s duration with an amplitude of 90% and 90 s pause between pulses. ChIP on the fragmented DNA was performed using the low cell # ChIP kit (no. kch-maglow-016, Diagenode, Belgium) and ChIP-grade monoclonal antibodies against ERα (no.Mab-009-050, Diagenode) or a control Ig (provided in the kit). The relative abundance of regions of interest in the precipitated DNA was quantitated by qPCR using SYBR Select PCR master mix (no. 4472903, Life Technologies, Carlsbad, CA, USA).

### Primers for ChIP qPCR

Bcl11b is a molecular partner of *Foxp3* and has been shown to be essential for T_reg_ cell’s suppressive function and maintenance of optimal *Foxp3* gene expression^49,62,63^. An *in silico* search revealed that the *BCL11B* promoter incorporates an ERE, hence we were able to design qPCR primers for this region to use as a known positive control gene for ChIP-qPCR in primary human T_reg_ cells. In contrast, our searches indicated that the *SETB1* promoter lacked any ERE, hence primers targeted against this gene served as a negative control (Supplementary Table 2).

### *FOXP3* gene

Based on our *in silico* analyses and published literature describing the *FOXP3* gene^22,42,64–68^, we designed quantitative PCR primers against: (a) Upstream region (−3888 to −3793 and −3665 to −3565); (b) tiling primers covering the region upstream of the core promoter (−943 to −664); (c) tiling primers covering the core promoter body (−456 to +104); (d) tiling primers covering CNS1 (+1593 to +1924); (e) tiling primers covering CNS2 (+3820 to +4051); (f) tiling primers covering CNS3 (+6443 to +6615). We then carried out pilot ChIP-qPCR analyses on blood Treg cells obtained from two healthy donors, followed by validation of the ER-enriched regions detected using ChIP-qPCR analyses of T_reg_ cells on a further cohort of six healthy donors. Primer sequences are listed in Supplementary Table 2.

### MCF7 cells

The *ERα-*expressing breast cancer cell line MCF7 was used as a positive control in the ERα ChIP-qPCR experiments. Primers to the ERE-containing gene *pS2*, and no ERE- containing *FKBP6* on chromosome 7 were used as positive and negative controls respectively (Supplementary Table 2)^69^.

### *In silico* identification of binding sites for ERα and interacting proteins

Position Specific Weight Matrices (PSWM) for the following Transcription Factor Binding Sites (TFBSs): ETS1, NFkB, RUNX1 and SMAD; and for complete ERE and ERE half sites were obtained using the Transfac tool (http://www.biobase-international.com/product/transcription-factor-binding-sites). The human promoter sequence for the *FOXP3* gene was obtained from the UCSC genome browser^70^. The TFBSs within regions of interest on the human *FOXP3* locus were identified using the matrices derived from Transfac and the Matrix-scan tool^71^ and the p-values assigned by the tool were recorded.

### Evolutionary conservation of *FOXP3* promoter regions

Regions of interest around the *FOXP3* gene in human, mouse and rat species were analysed using both the Evolutionary Conserved Regions (ECR) browser^72^ and the MULAN tool^73^ to identify similar TFBS patterns between species. The latter tool revealed the conserved regions (sequences shown in Figs. S8–S11). The conserved TFBSs were manually identified and marked in the output file. The ECR browser provided a graphical summary of the sequences studied and the corresponding human genome (top portions in Figs. S8–S11). Evolutionary conserved regions were identified based on sequence similarity between species (assessed in ECR browser). Sequences were aligned by optimizing alignment identity scores and a threshold of 50% sequence homology per 100 base block was used to define conservation (performed using the MULAN tool in the ECR browser).

### Statistics

All tests were performed in triplicate. Significant differences between groups were determined using either student’s t-test or paired t-tests as appropriate (Graph Pad Prism 5 Software, San Diego, CA, USA). P-values < 0.05 were considered significant (*P < 0.05; **P < 0.01; ***P < 0.001; ****P < 0.0001).

### Study approval

Written informed consent was obtained from all study participants prior to inclusion in the study. The study was approved by Institutional Medical Ethics Committee of Kidwai Memorial Institute of Oncology (vide letter no. PER/CAB-I/D-1-13/2006).

## Electronic supplementary material

Supplementary files

## References

[1] Sakaguchi S (2006). Foxp3+CD25+CD4+ natural regulatory T cells in dominant self-tolerance and autoimmune disease. Immunol. Rev..

[2] Ochs HD, Ziegler SF, Torgerson TR (2005). FOXP3 acts as a rheostat of the immune response. Immunol. Rev..

[3] Burzyn D, Benoist C, Mathis D (2013). Regulatory T cells in nonlymphoid tissues. Nat. Immunol..

[4] Yang XF (2008). Factors regulating apoptosis and homeostasis of CD4+CD25 (high) FOXP3+ regulatory T cells are new therapeutic targets. Front. Biosci..

[5] Hoeppli RE, Wu D, Cook L, Levings MK (2015). The environment of regulatory T cell biology: cytokines, metabolites, and the microbiome. Front. Immunol..

[6] Polanczyk MJ (2004). Cutting edge: Estrogen drives expansion of the CD4+CD25+ regulatory T cell compartment. J. Immunol..

[7] Polanczyk MJ, Hopke C, Vandenbark AA, Offner H (2006). Estrogen-mediated immunomodulation involves reduced activation of effector T cells, potentiation of Treg cells, and enhanced expression of the PD-1 costimulatory pathway. J. Neurosci. Res..

[8] Prieto GA, Rosenstein Y (2006). Oestradiol potentiates the suppressive function of human CD4+CD25+ regulatory T cells by promoting their proliferation. Immunology.

[9] Tai P (2008). Induction of regulatory T cells by physiological level estrogen. J. Cell. Physiol..

[10] Valor L (2011). Estradiol-dependent perforin expression by human regulatory T-cells. Eur. J. Clin. Invest..

[11] Yates MA, Li Y, Chlebeck PJ, Offner H (2010). GPR30, but not estrogen receptor-alpha, is crucial in the treatment of experimental autoimmune encephalomyelitis by oral ethinyl estradiol. BMC Immunol..

[12] Luo CY, Wang L, Sun C, Li DJ (2011). Estrogen enhances the functions of CD4(+)CD25(+)Foxp3(+) regulatory T cells that suppress osteoclast differentiation and bone resorption *in vitro*. Cell. Mol. Immunol..

[13] Dowsett M, Folkerd E (2005). Deficits in plasma oestradiol measurement in studies and management of breast cancer. Breast Cancer Res..

[14] Polanczyk MJ, Hopke C, Huan J, Vandenbark AA, Offner H (2005). Enhanced FoxP3 expression and Treg function in pregnant and estrogen-treated mice. J. Neuroimmunol..

[15] Osborne CK, Wakeling A, Nicholson RI (2004). Fulvestrant: an oestrogen receptor antagonist with a novel mechanism of action. Br. J. Cancer..

[16] Robertson JF (2004). Pharmacokinetic profile of intramuscular fulvestrant in advanced breast cancer. Clin. Pharmacokinet..

[17] Adurthi S (2012). Functional tumor infiltrating TH1 and TH2 effectors in large early-stage cervical cancer are suppressed by regulatory T cells. Int. J. Gynecol. Cancer..

[18] Mason CE (2010). Location analysis for the estrogen receptor-alpha reveals binding to diverse ERE sequences and widespread binding within repetitive DNA elements. Nucleic Acids Res..

[19] Anderson I, Gorski J (2000). Estrogen receptor alpha interaction with estrogen response element half-sites from the rat prolactin gene. Biochemistry..

[20] Zheng Y (2010). Role of conserved non-coding DNA elements in the Foxp3 gene in regulatory T-cell fate. Nature..

[21] Ito K (2007). Hormone replacement therapy and cancers: the biological roles of estrogen and progestin in tumorigenesis are different between the endometrium and breast. Tohoku J. Exp. Med..

[22] Lønning PE (2011). Exploring breast cancer estrogen disposition: the basis for endocrine manipulation. Clin. Cancer Res..

[23] Nair HB (2005). Induction of aromatase expression in cervical carcinomas: effects of endogenous estrogen on cervical cancer cell proliferation. Cancer Res..

[24] Moodley M, Moodley J, Chetty R, Herrington CS (2003). The role of steroid contraceptive hormones in the pathogenesis of invasive cervical cancer: a review. Int. J. Gynecol. Cancer..

[25] Arbeit JM, Münger K, Howley PM, Hanahan D (1994). Progressive squamous epithelial neoplasia in K14-human papillomavirus type 16 transgenic mice. J. Virol..

[26] Chung SH, Shin MK, Korach KS, Lambert PF (2013). Requirement for stromal estrogen receptor alpha in cervical neoplasia. Horm. Cancer..

[27] Kwasniewska A (2011). Estrogen and progesterone receptor expression in HPV-positive and HPV-negative cervical carcinomas. Oncol. Rep. Oncol. Rep..

[28] den Boon JA (2015). Molecular transitions from papillomavirus infection to cervical precancer and cancer: Role of stromal estrogen receptor signaling. Proc. Natl. Acad. Sci. USA.

[29] Adurthi S (2008). Regulatory T Cells in a Spectrum of HPV-Induced Cervical Lesions: Cervicitis, Cervical Intraepithelial Neoplasia and Squamous Cell Carcinoma. Am. J. Reprod. Immunol..

[30] Aristimuño C (2012). Sex-hormone receptors pattern on regulatory T-cells: clinical implications for multiple sclerosis. Clin. Exp. Med..

[31] Lee JH, Ulrich B, Cho J, Park J, Kim CH (2011). Progesterone promotes differentiation of human cord blood fetal T cells into T regulatory cells but suppresses their differentiation into Th17 cells. J Immunol..

[32] Hori S, Nomura T, Sakaguchi S (2003). Control of regulatory T cell development by the transcription factor Foxp3. Science.

[33] Fontenot JD, Gavin MA, Rudensky AY (2003). Foxp3 programs the development and function of CD4+CD25+ regulatory T cells. Nature Immunol..

[34] Bacchetta R (2006). Defective regulatory and effector T cell functions in patients with FOXP3 mutations. J. Clin. Invest..

[35] Filaci G (2007). CD8+CD28- T regulatory lymphocytes inhibiting T cell proliferative and cytotoxic functions infiltrate human cancers. J. Immunol..

[36] Sekiya T (2011). The nuclear orphan receptor Nr4a2 induces Foxp3 and regulates differentiation of CD4+ T cells. Nat. Commun..

[37] Okada M, Hibino S, Someya K, Yoshmura A (2014). Regulation of regulatory T cells: epigenetics and plasticity. Adv. Immunol..

[38] Ruan Q (2009). Development of Foxp3 (+) regulatory t cells is driven by the c-Rel enhanceosome. Immunity.

[39] Polansky JK (2010). Methylation matters: binding of Ets-1 to the demethylated Foxp3 gene contributes to the stabilization of Foxp3 expression in regulatory T cells. J. Mol. Med. (Berl)..

[40] Ogawa C (2014). TGF-β-mediated Foxp3 gene expression is cooperatively regulated by Stat5, Creb, and AP-1 through CNS2. J. Immunol..

[41] Long M, Park SG, Strickland I, Hayden MS, Ghosh S (2009). Nuclear factor-kappaB modulates regulatory T cell development by directly regulating expression of Foxp3 transcription factor. Immunity..

[42] Ouyang W (2010). Foxo proteins cooperatively control the differentiation of Foxp3+ regulatory T cells. Nat. Immunol..

[43] Morelli C (2010). Akt2 inhibition enables the forkhead transcription factor FoxO3a to have a repressive role in estrogen receptor alpha transcriptional activity in breast cancer cells. Mol. Cell. Biol..

[44] Stender JD (2010). Genome-wide analysis of estrogen receptor alpha DNA binding and tethering mechanisms identifies Runx1 as a novel tethering factor in receptor-mediated transcriptional activation. Mol. Cell. Biol..

[45] Hirano S, Furutama D, Hanafusa T (2007). Physiologically high concentrations of 17 beta-estradiol enhance NF-kappaB activity in human T cells. Am. J. Physiol. Regul. Integr. Comp. Physiol..

[46] Feng Y (2014). Control of the inheritance of regulatory T cell identity by a cis element in the Foxp3 locus. Cell.

[47] Li X, Liang Y, LeBlanc M, Benner C, Zheng Y (2014). Function of a Foxp3 cis-element in protecting regulatory T cell identity. Cell.

[48] Gavin MA (2007). Foxp3-dependent programme of regulatory T-cell differentiation. Nature.

[49] Rudra D (2012). Transcription factor Foxp3 and its protein partners form a complex regulatory network. Nat. Immunol..

[50] Acevedo ML, Kraus WL (2003). Mediator and p300/CBP-steroid receptor co-activator complexes have distinct roles, but function synergistically, during estrogen receptor alpha-dependent transcription with chromatin templates. Mol. Cell. Biol..

[51] Liu Y (2014). Two Histone/Protein Acetyl transferases, CBP and p300, are indispensable for Foxp3+ T-Regulatory Cell Development and Function. Mol. Cell. Biol..

[52] Fullwood MJ (2009). An oestrogen-receptor-α-bound human chromatin interactome. Nature.

[53] Kumar MM (2016). Role of estrogen receptor alpha in human cervical cancer-associated fibroblasts: a transcriptomic study. Tumour Biol..

[54] Polese B (2014). The Endocrine Milieu and CD4 T Lymphocyte Polarization during Pregnancy. Front Endocrinol (Lausanne)..

[55] Rao AJ, Kotagi SG, Moudgal NR (1984). Serum concentrations of chorionic gonadotrophin, oestradiol-17 beta and progesterone during early pregnancy in the south Indian bonnet monkey (Macaca radiata). J. Reprod. Fertil..

[56] Kocanova S, Mazaheri M, Subra SC, Bystricky K (2010). Ligands specify estrogen receptor alpha nuclear localization and degradation. BMC Cell Biology.

[57] Ishikawa H (2009). High aromatase expression in uterine leiomyoma tissues of African-American women. J Clin Endocrinol Metab.

[58] Dulkys Y (2001). Detection of mRNA for eotaxin-2 and eotaxin-3 in human dermal fibroblasts and their distinct activation profile on human eosinophils. J Invest Dermatol..

[59] Morgan ME (2005). Expression of FOXP3 mRNA is not confined to CD4+CD25+ T regulatory cells in humans. Hum Immunol..

[60] Matsuzaki S (2001). Expression of estrogen receptor alpha and beta in peritoneal and ovarian endometriosis. Fertil Steril..

[61] Zhang Y (2007). PGC-1alpha induces apoptosis in human epithelial ovarian cancer cells through a PPARgamma-dependent pathway. Cell Res..

[62] Vanvalkenburgh J (2011). Critical role of Bcl11b in suppressor function of T regulatory cells and prevention of inflammatory bowel disease. J. Exp. Med..

[63] Fu W (2012). A multiply redundant genetic switch ‘locks in’ the transcriptional signature of regulatory T cells. Nat. Immunol..

[64] Mantel PY (2006). Molecular mechanisms underlying FOXP3 induction in human T cells. J. Immunol..

[65] Kim HP, Leonard WJ (2007). CREB/ATF-dependent T cell receptor induced FoxP3 gene expression: a role for DNA methylation. J. Exp. Med..

[66] Burchill MA, Yang J, Vogtenhuber C, Blazar BR, Farrar MA (2007). IL-2 receptor beta-dependent STAT5 activation is required for the development of Foxp3+ regulatory T cells. J. Immunol..

[67] Klunker S (2009). Transcription factors RUNX1 and RUNX3 in the induction and suppressive function of Foxp3+ inducible regulatory T cells. J. Exp. Med..

[68] Barbarulo A (2011). Notch3 and canonical NF-kappaB signaling pathways cooperatively regulate Foxp3 transcription. J. Immunol..

[69] Wang C, Yu J, Kallen CB (2008). Two estrogen response element sequences near the PCNA gene are not responsible for its estrogen enhanced expression in MCF7 cells. PLoS One..

[70] Kent WJ (2002). The human genome browser at UCSC. Genome Res..

[71] Turatsinze JV, Thomas-Chollier M, Defrance M, van Helden J (2008). Using RSAT to scan genome sequences for transcription factor binding sites and cis-regulatory modules. Nat. Protoc..

[72] Ovcharenko I, Nobrega MA, Loots GG, Stubbs L (2004). ECR Browser: a tool for visualizing and accessing data from comparisons of multiple vertebrate genomes. Nucleic Acids Res..

[73] Ovcharenko I (2005). Multiple-sequence local alignment and visualization for studying function and evolution. Genome Res..

