# Supplementary material for: Oestrogen Receptor-α binds the FOXP3 promoter and modulates regulatory T-cell function in human cervical cancer
Source: Sci Rep. 2017 Dec 11;7:17289. doi: 10.1038/s41598-017-17102-w (PMC5725534; doi:10.1038/s41598-017-17102-w)
Supplement: Supplementary file 1 — Supplementary files [file 41598_2017_17102_MOESM1_ESM.pdf]

Oestrogen Receptor-α binds the *FOXP3* promoter and modulates regulatory T-cell function in human cervical cancer

Authors: Sreenivas Adurthi, Mahesh M Kumar, H.S. Vinodkumar, Geetashree Mukherjee, H. Krishnamurthy, Acharya Kshitish K, U. D. Bafna, Devi K. Uma, Abhishekh B, Sudhir Krishna, Parchure A, Alka Murali, and R. S. Jayshree

Corresponding author:
*R. S. Jayshree,
Prof. and Head, Department of Microbiology,
R. No. 114, 1st Floor, Main Block,
Kidwai Memorial Institute of Oncology,
Dr. M.H. Marigowda Road,
Bangalore – 560029. India.
91-80-26573429
91-80-26094073/74
Fax: 91-80-26560723
Email: microjayshree@gmail.com

ERα

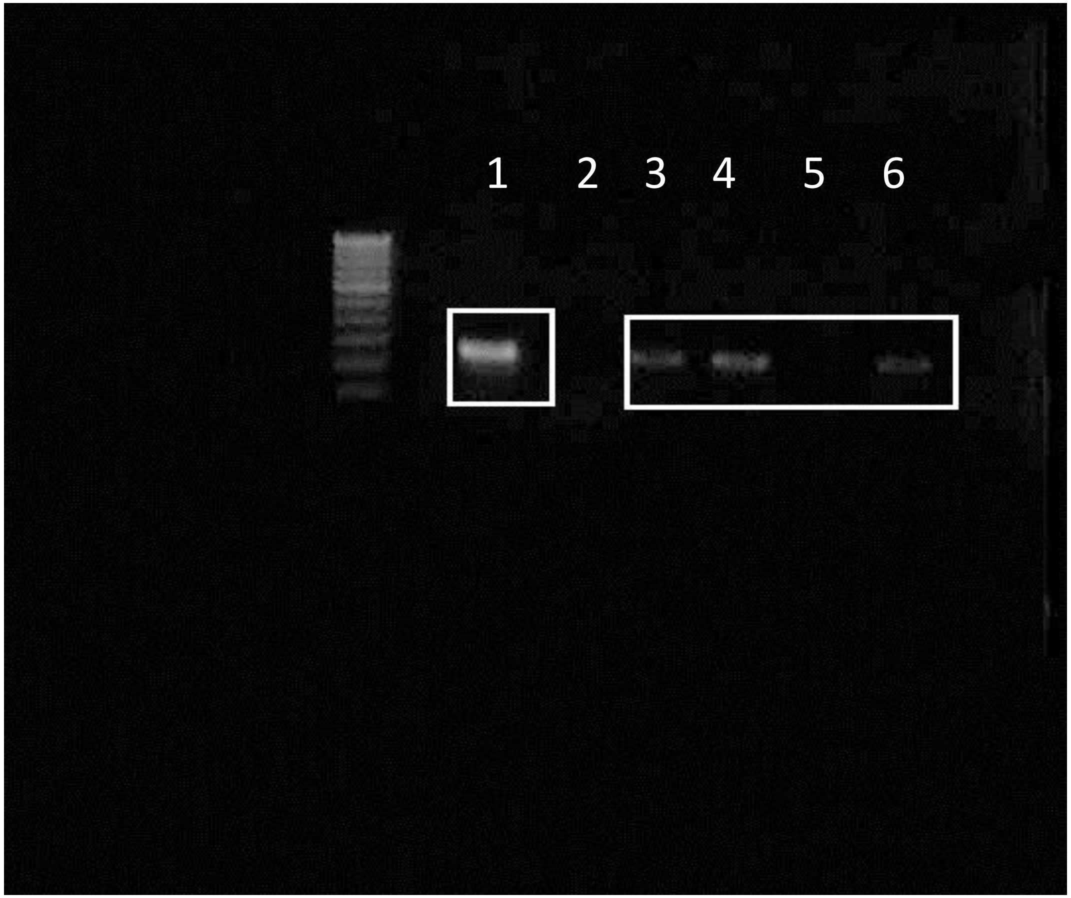

Fig. S1a: *Original gel picture of* **Fig. 2A.** *ERα expression in T cell subsets: 1: MCF; 2: TNbp; 3: TRt; 4: TEt; 5: TNt; 6: TRbd (male). Cropping lines are indicated.*

ERα

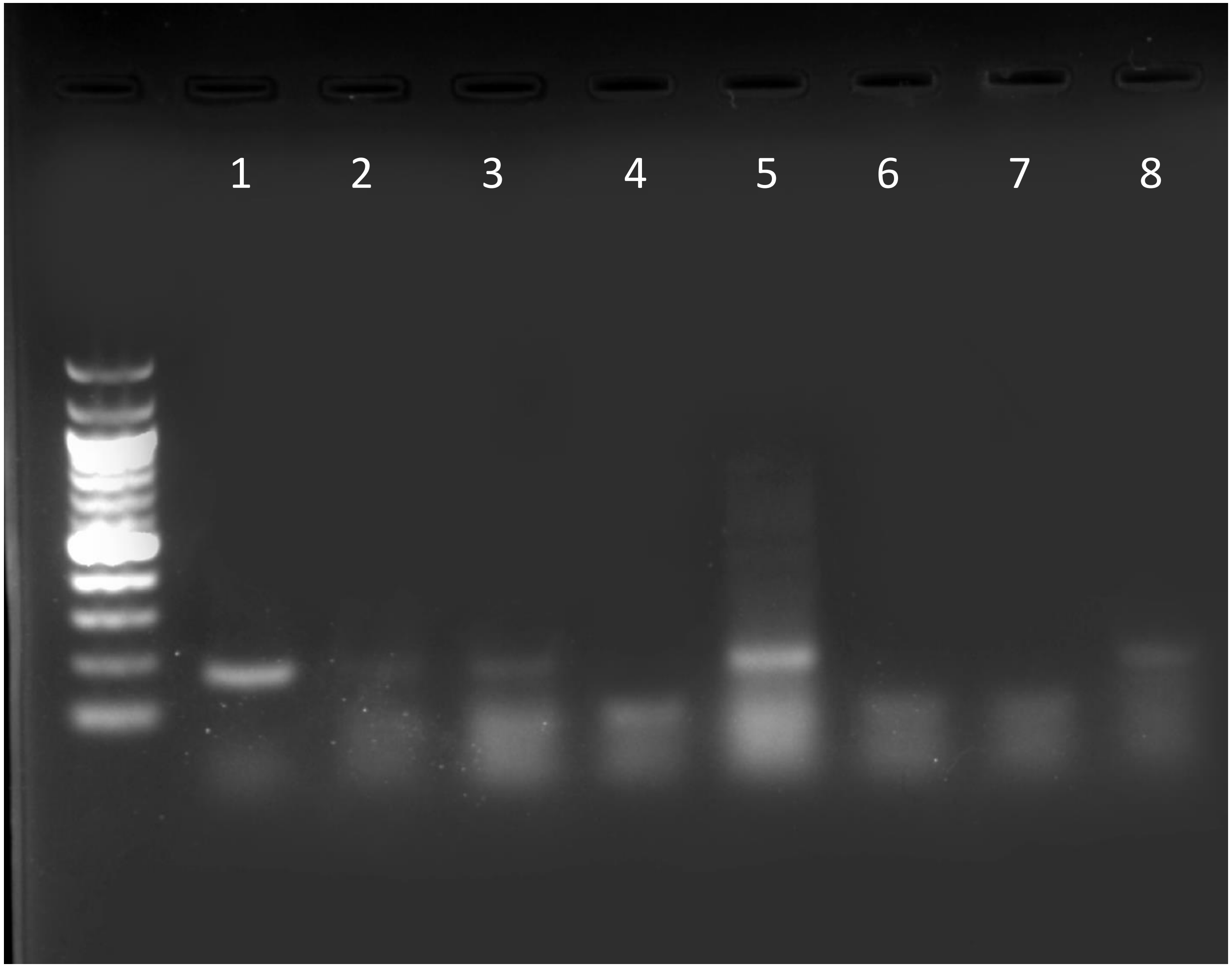

Fig. S1b: *ERα expression in T cell subsets: Lanes: 1. MCF; 2: CD8+t; 3. TEt; 4. TNt; 5: TRbd (male); 6: TNbd (male);7: TNbp; 8: TRt*

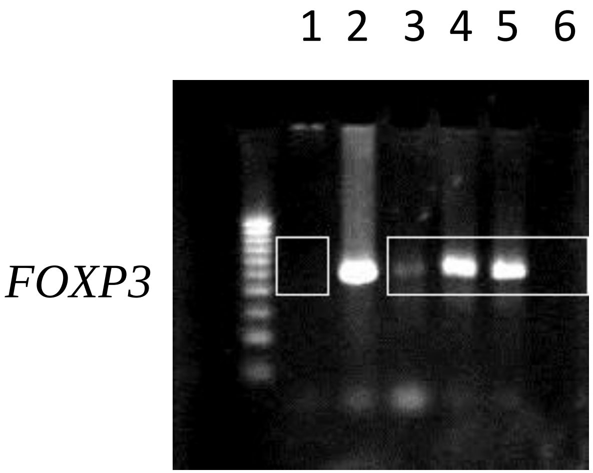

Fig. S2a: *Original gel picture of Fig. 2A. FOXP3 expression in T cell subsets. Lanes: 1: TNbp; 2 TRbd (male); 3: TEt; 4: TRt; 5: MCF; 6: TNt. Cropping lines are indicated.*

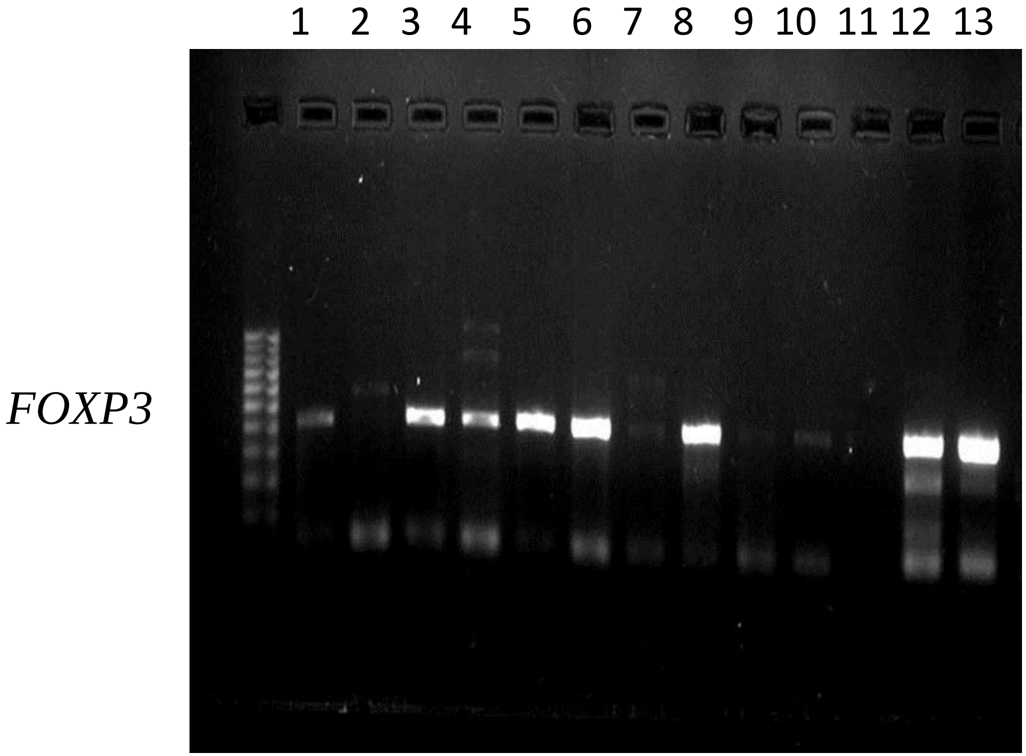

Fig. S2b: *FOXP3 expression in T cell subsets. Lanes: 1:TEt; 2: TNt; 3: TRbd (male); 4 :TRbp; 5:TRt ; 6:TRbd (female); 7: TEbd (male); 8: MCF; 9:TEbp; 10:CD8+t; 11: CD8+bp; 12 and 13: FOXP3 plasmids.*

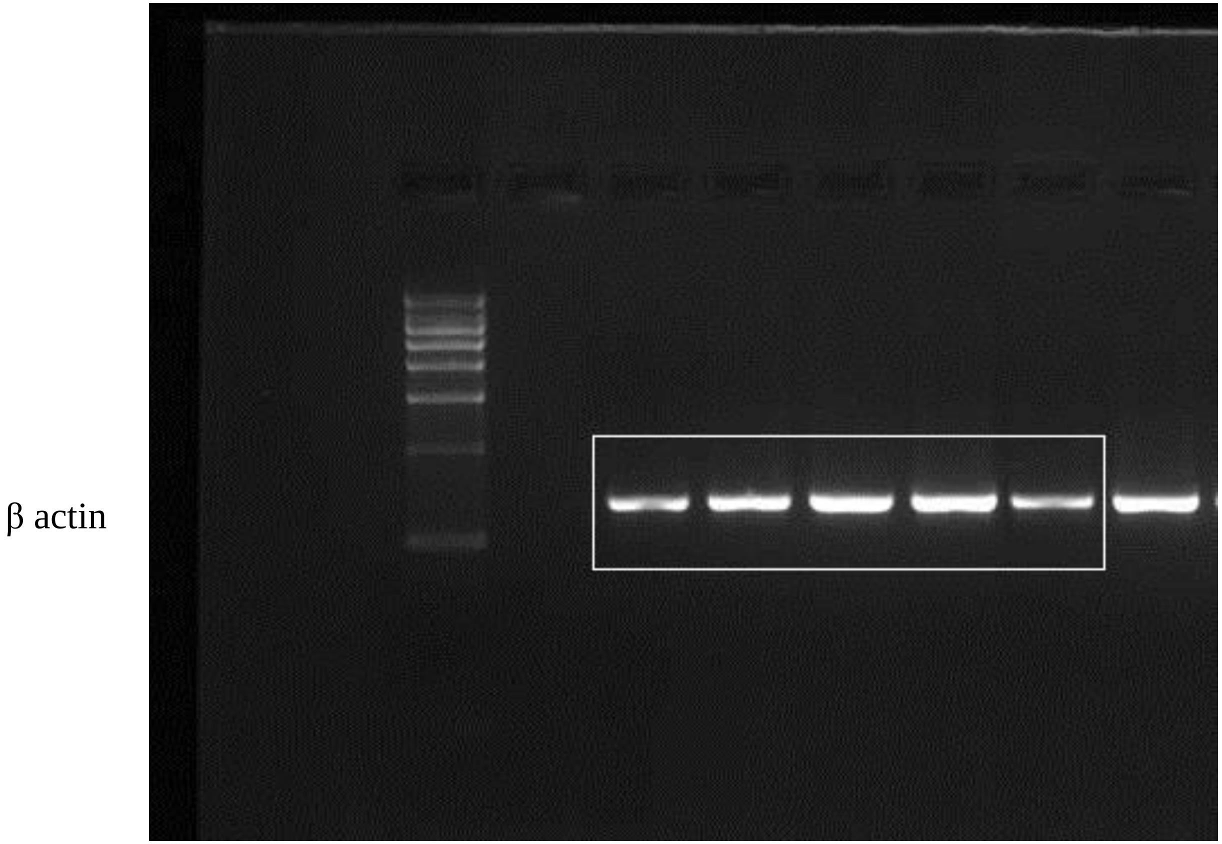

Fig. S3: *Original gel picture of Fig. 2A. Beta actin expression in T cell subsets. Cropping lines are indicated.*

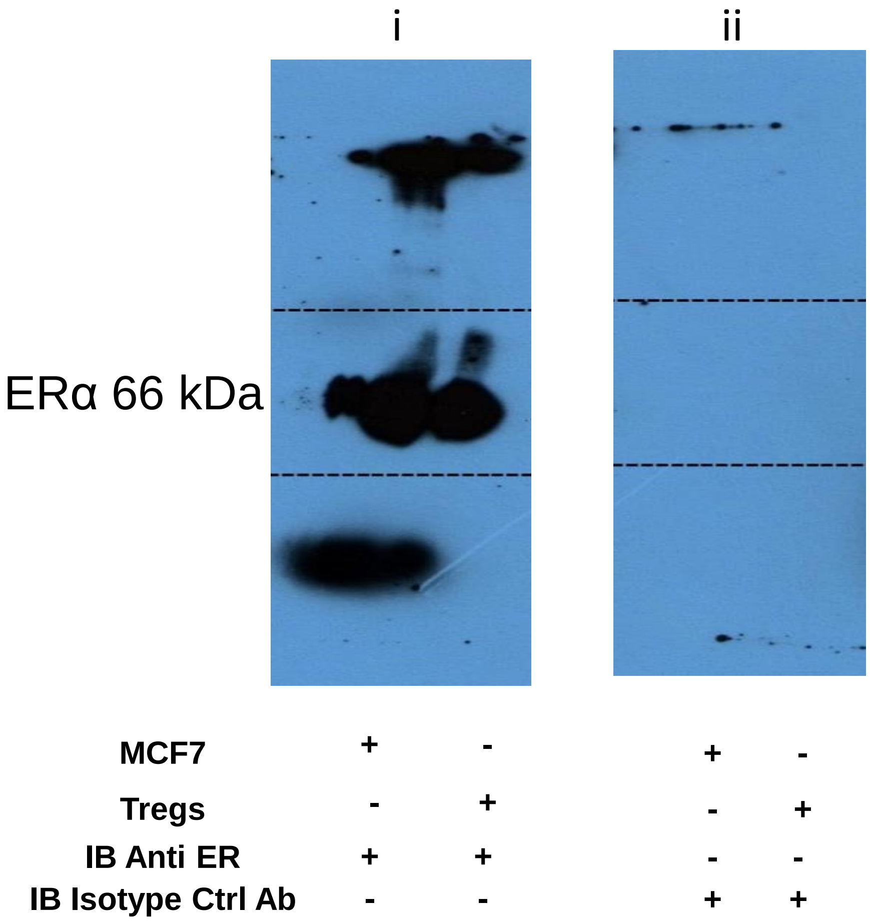

Fig.S4: Original blot of fig. 2C. *Estradiol complexes present in MACS/sort-purified tumour T_{reg} cells as detected by immunoprecipitation of estradiol complexes using anti-E2 antibodies followed by immunoblotting with anti-ERα antibodies (i) or isotype-matched control antibodies (ii). Data are representative of 6 independent experiments. Gels have been run under the same experimental conditions. Though multiple immunoreactive bands of ERα were seen in both MCF and Tregs, which was lost after ICI treatment, for the sake of brevity, only the 66 kDa band has been depicted in fig. 2C. Cropping lines are indicated.*

β actin

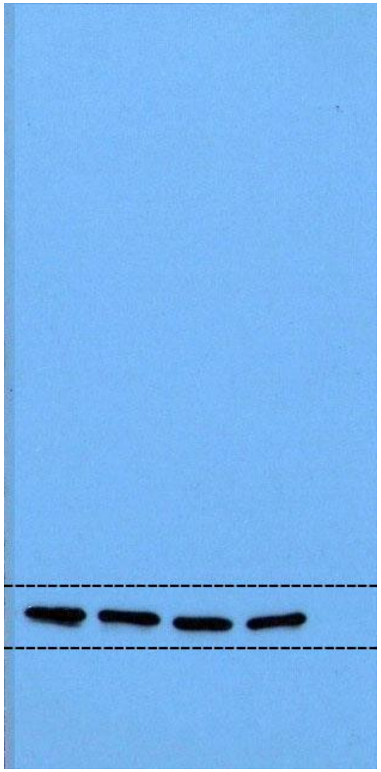

MCF7	+	-	+	-
Tregs	-	+	-	+
IB Anti ER	+	+	-	-
IB Isotype Ctrl Ab	-	-	+	+

Fig.S5: Original blot of fig. 2C. *Beta actin expression in Tregs cells. IP / IB of Treg cells with anti β actin antibodies. Cropping lines are indicated.*

ERα

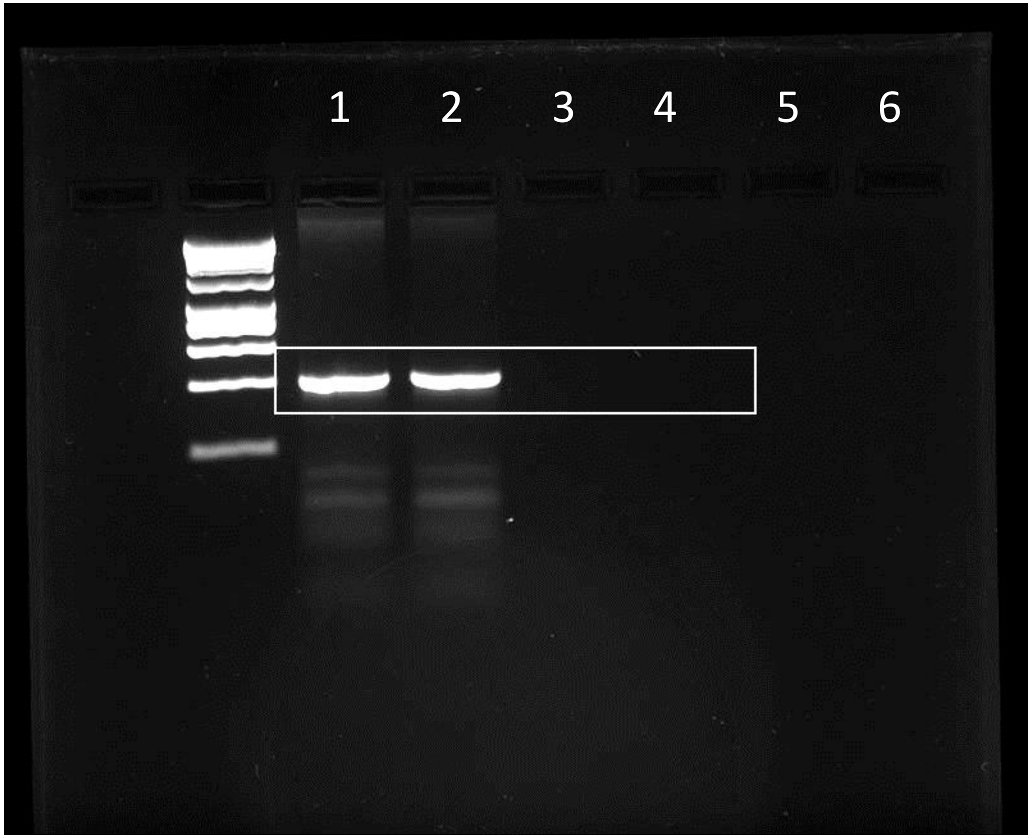

MCF7	+	-	-	-	-	-
Tregs	-	+	+	+	+	+
ICI 1.8uM	-	-	+	+	-	-
ICI 36.2nM	-	-	-	-	+	+
E2 3.6nM	-	-	-	+	-	+

Fig. S6: *Original gel of Fig. 3A. ERα expression in Treg cells after treatment or not with the ERα antagonist ICI 182,780 (ICI; 1.8µM and 36.2nM) for 72h in the presence or absence of exogenous estradiol (E2:3.6nM). Cropping lines are indicated.*

1 2 3 4 5 6

FOXP3

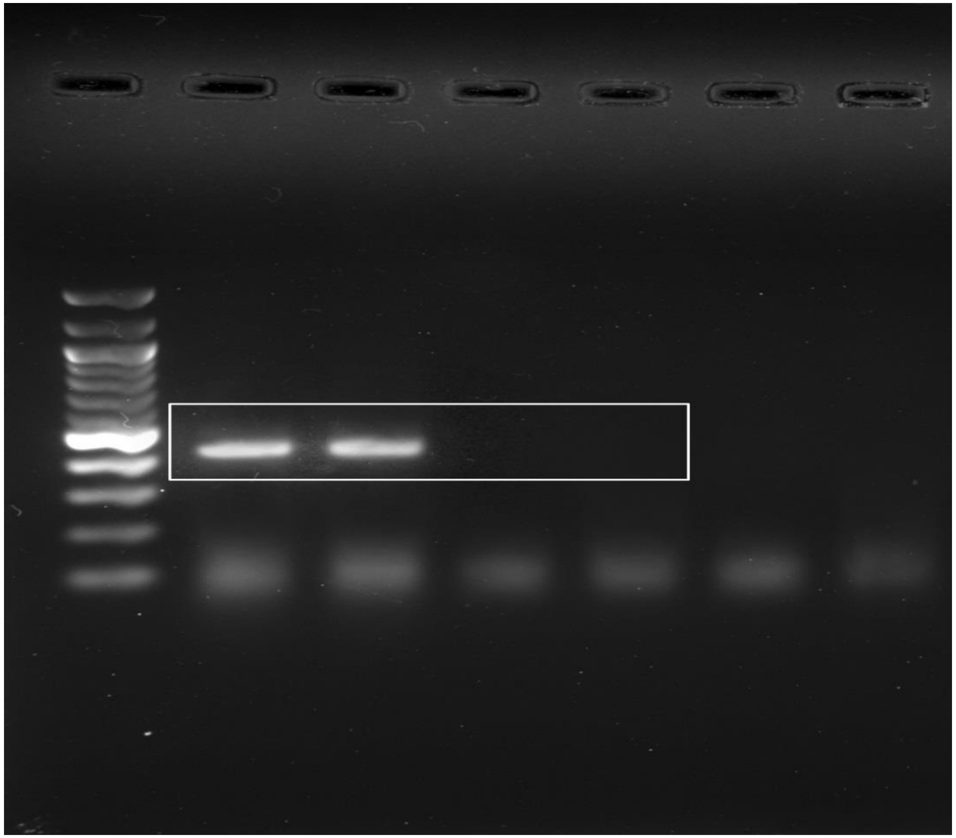

	1	2	3	4	5	6
MCF7	+	-	-	-	-	-
Tregs	-	+	+	+	+	+
ICI 1.8uM	-	-	+	+	-	-
ICI 36.2nM	-	-	-	-	+	+
E2 3.6nM	-	-	-	+	-	+

Fig. S7: *Original gel of Fig. 3A. FOXP3 expression in Treg cells after treatment or not with the ERα antagonist ICI 182,780 (ICI; 1.8μM and 36.2nM) for 72h in the presence or absence of exogenous estradiol (E2:3.6nM). Cropping lines are indicated.*

β actin

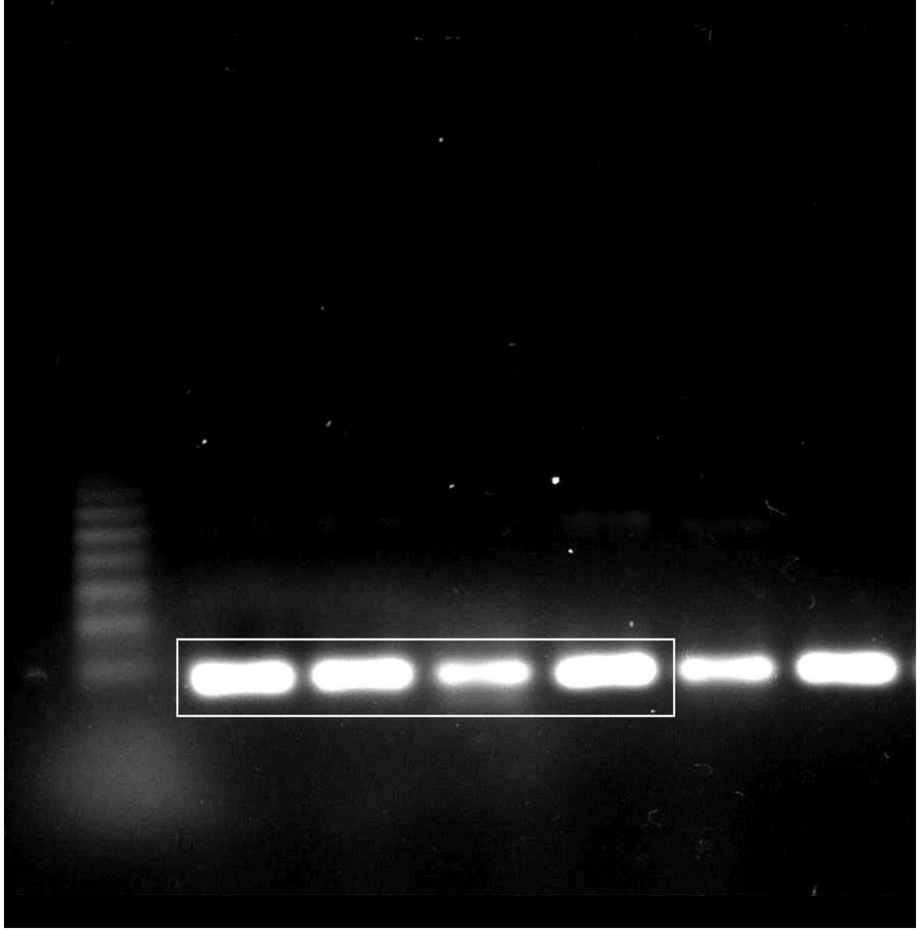

MCF7	+	-	-	-	-	-
Tregs	-	+	+	+	+	+
ICI 1.8uM	-	-	+	+	-	-
ICI 36.2nM	-	-	-	-	+	+
E2 3.6nM	-	-	-	+	-	+

***Fig. S8**: Original gel of Fig. 3A. Beta actin expression in Treg cells after treatment or not with the ERα antagonist ICI 182,780 (ICI; 1.8μM and 36.2nM) for 72h in the presence or absence of exogenous estradiol (E2:3.6nM). Cropping lines are indicated.*

ERα 66 kDa

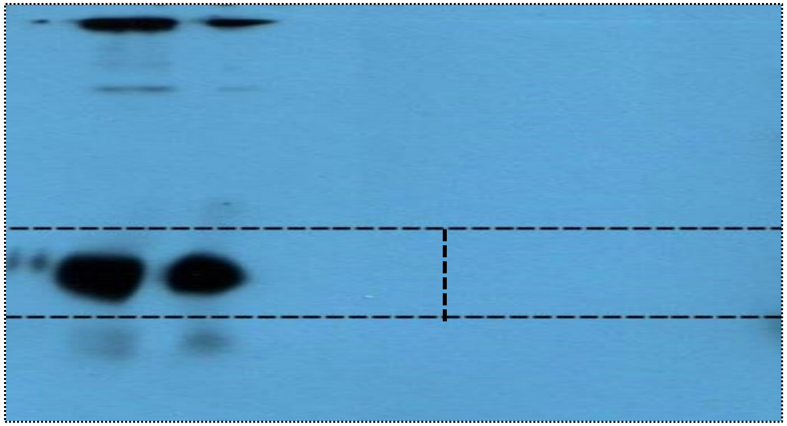

MCF	+	-	-	-	-	-	-	-
Tregs	-	+	+	+	+	+	+	+
ICI 1.8µM	-	-	+	+	-	-	-	-
ICI 362nM	-	-	-	-	+	+	-	-
ICI 36.2nM	-	-	-	-	-	-	+	+
E2 3.6nM	-	-	-	+	-	+	-	+

Fig. S9: *Original blot picture of Fig.3C . ERα expression in Treg cells after treatment or not with the ERα antagonist ICI 182,780 (ICI; 1.8µM and 36.2nM) for 72h in the presence or absence of exogenous estradiol (E2:3.6nM). IP/IB of Treg cells with ERα antibodies. Cropping lines are indicated.*

β Actin

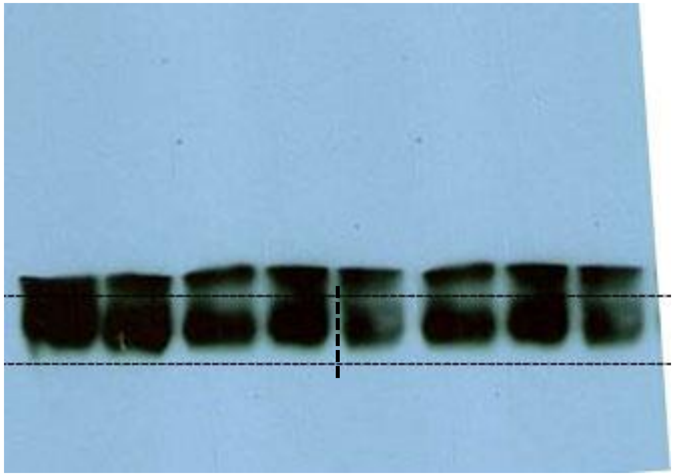

MCF	+	-	-	-	-	-	-	-
Tregs	-	+	+	+	+	+	+	+
ICI 1.8µM	-	-	+	+	-	-	-	-
ICI 362nM	-	-	-	-	+	+	-	-
ICI 36.2nM	-	-	-	-	-	-	+	+
E2 3.6nM	-	-	-	+	-	+	-	+

Fig. S10 : *Original blot picture of Fig. 3C. Beta actin expression in Treg cells. Dotted lines indicate cropping position. IP /IB of Treg cells with anti β actin* antibodies.

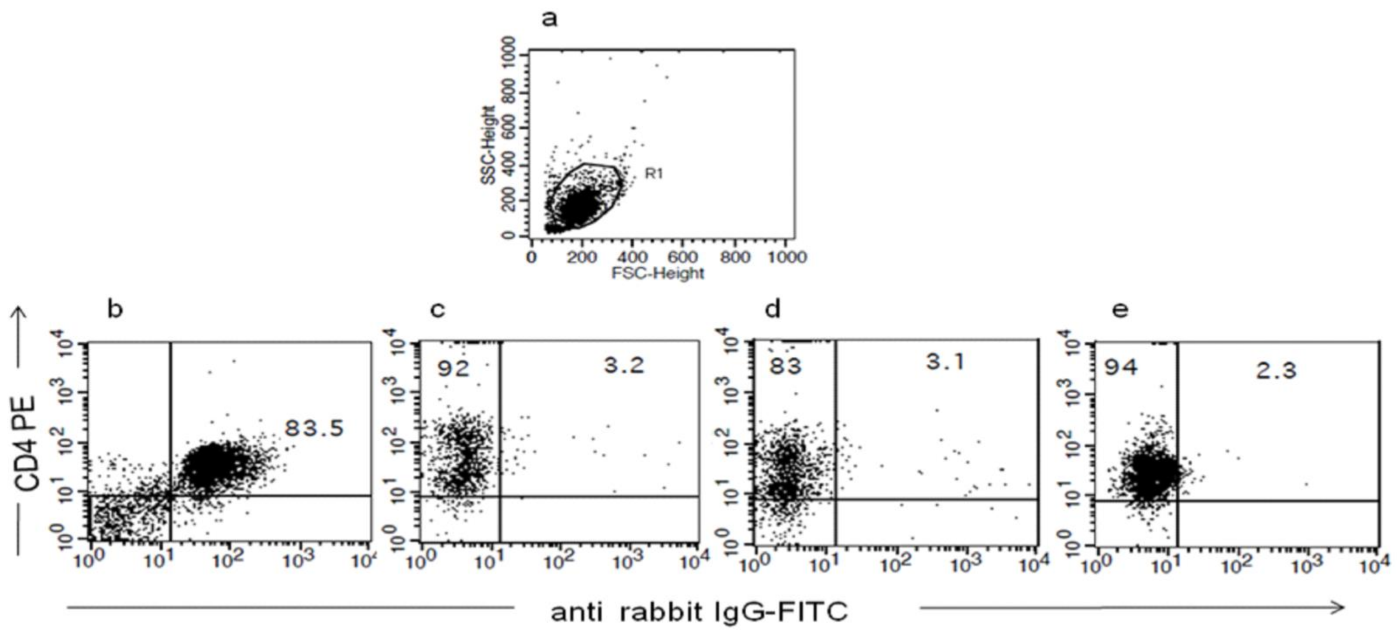

Fig. S11: *Effect of ICI treatment on ERα expression in tumor T_{regs} cells as measured by flow-cytometry: CxCa-derived T_{reg} cells were stained with rabbit anti-human ERα antibodies (or isotype control) followed by staining with FITC-labelled goat anti-rabbit IgG. Dot plots show light scatter gating of MACS-enriched T_{reg} cells (a) and ERα expression in T_{reg} cells that were either left untreated (b) or exposed to low (c) medium (d) or high (e) concentrations of inhibitor ICI (respectively 36.2nM, 362nM, and 1.8µM). Data are representative of 6 independent experiments.*

FOXP3 48 kDa

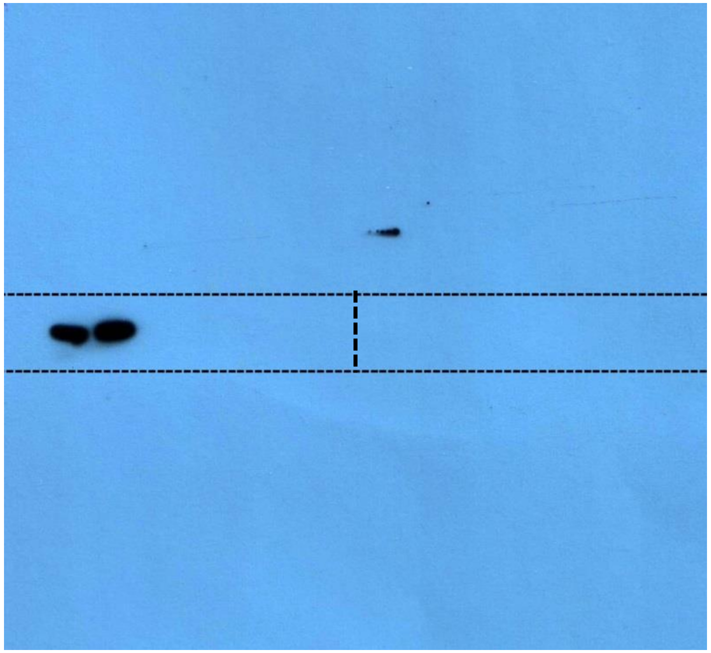

MCF	+	-	-	-	-	-	-	-
Tregs	-	+	+	+	+	+	+	+
ICI 1.8µM	-	-	+	+	-	-	-	-
ICI 362nM	-	-	-	-	+	+	-	-
ICI 36.2nM	-	-	-	-	-	-	+	+
E2 3.6nM	-	-	-	+	-	+	-	+

Fig. S12: *Original blot picture of Fig. 3D. FOXP3 expression in Treg cells. IP/IB of Treg cells with anti FOXP3 antibodies. Dotted lines indicate cropping position.*

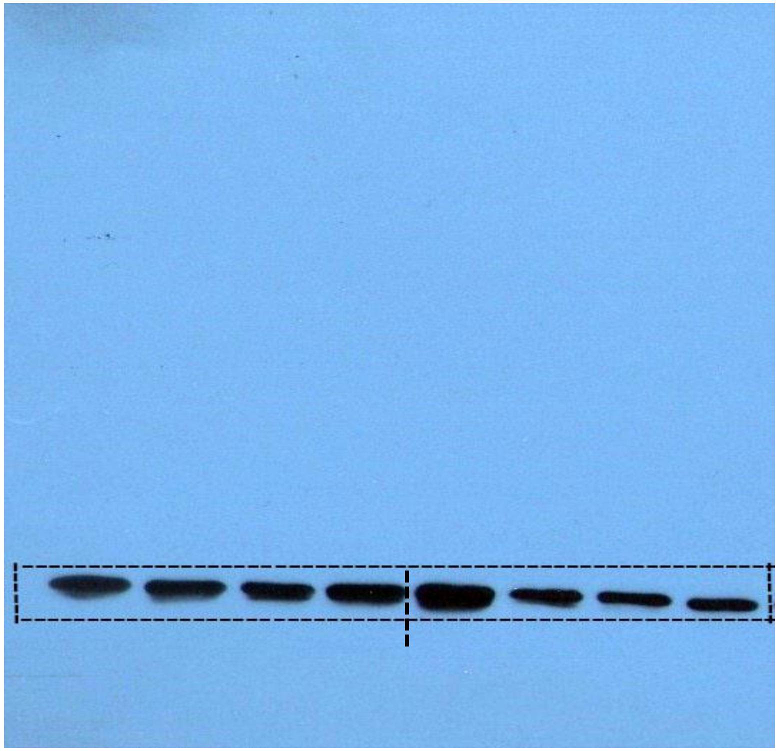

β Actin

MCF	+	-	-	-	-	-	-	-
Tregs	-	+	+	+	+	+	+	+
ICI 1.8µM	-	-	+	+	-	-	-	-
ICI 362nM	-	-	-	-	+	+	-	-
ICI 36.2nM	-	-	-	-	-	-	+	+
E2 3.6nM	-	-	-	+	-	+	-	+

Fig. S13: *Original blot picture of Fig. 3D. Beta actin expression in Treg cells. IP IB with anti beta actin antibodies. Dotted lines indicate cropping position.*

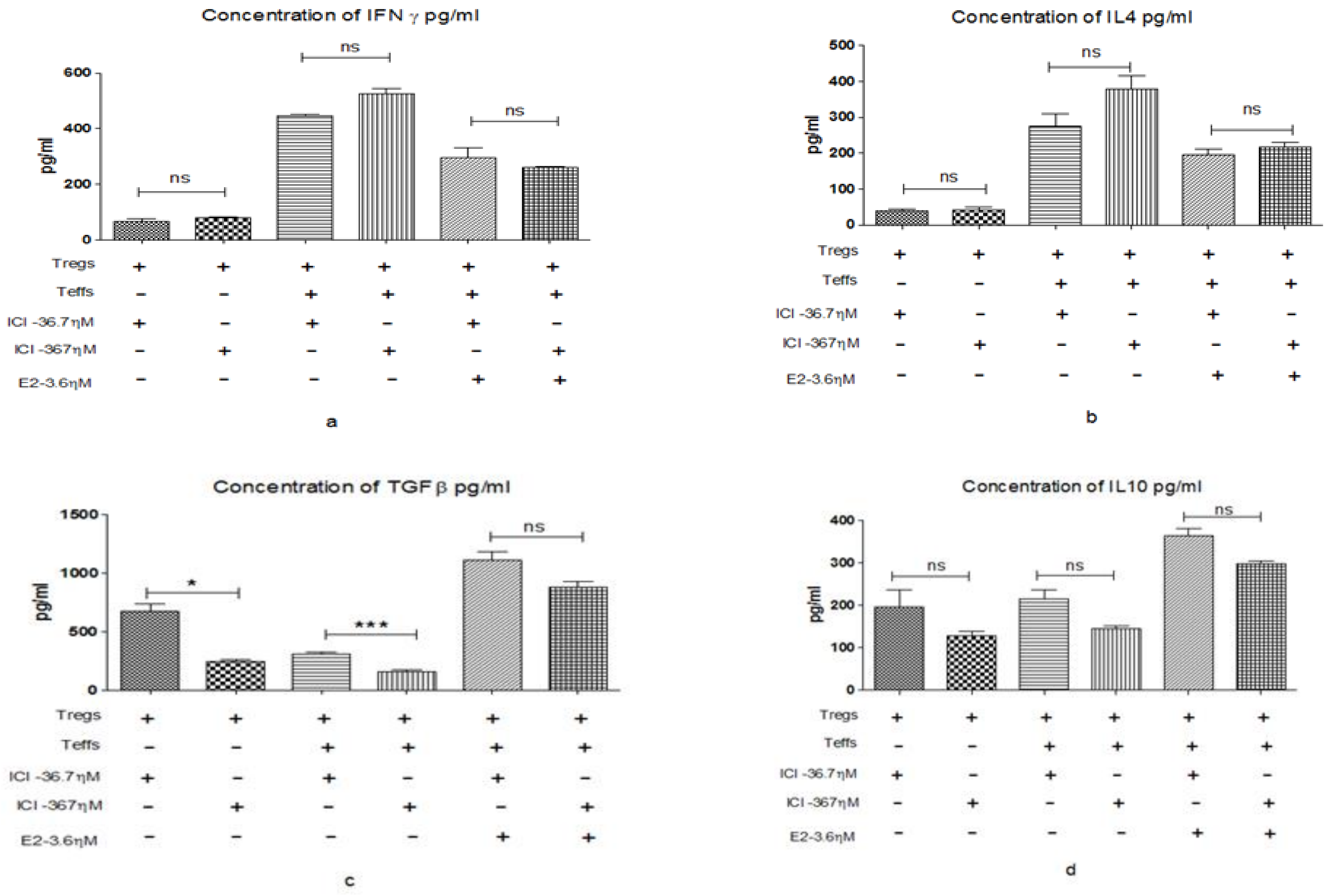

Fig.S14: *Effect of ICI-treated T_{reg} cells on cytokine production by co-cultured T_{eff} cells. Upon ICI treatment, we observed a dose-dependent decrease in TGFβ production by CxCa-derived T_{reg} cells either in the presence or absence of autologous T_{eff} cells.*

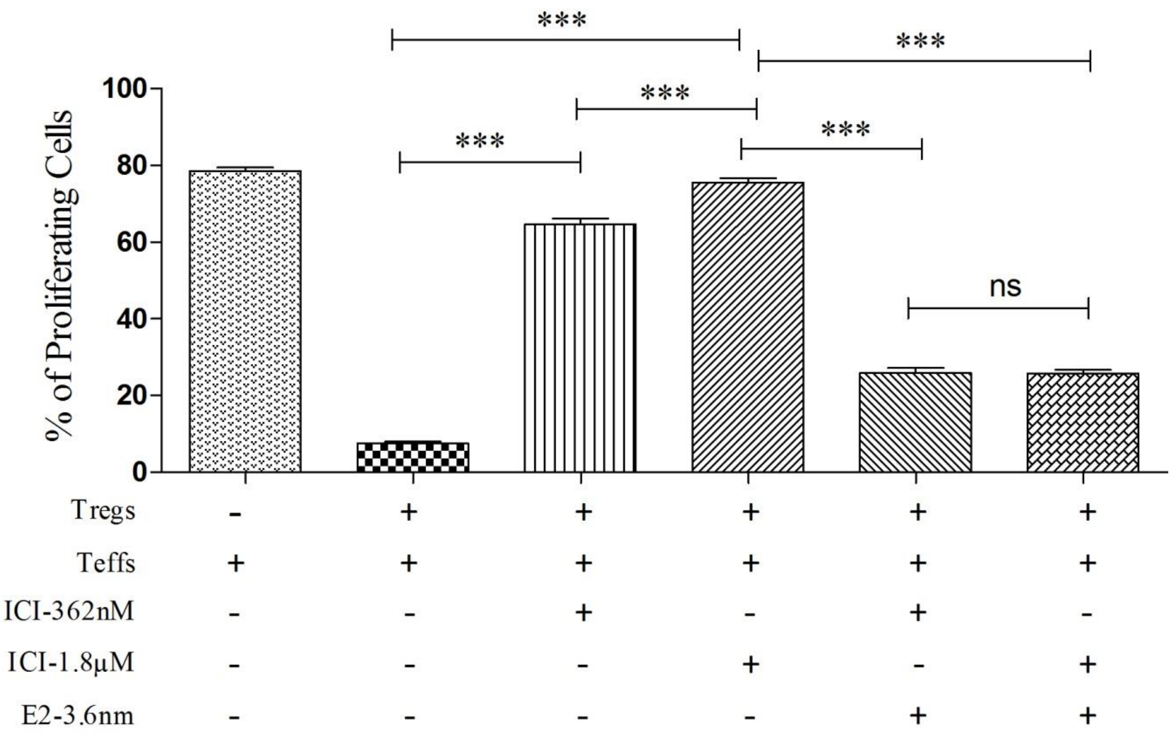

Fig.S15: *Effect of graded concentrations of ICI and E2 treatment on suppressive function of T_{reg} cells – evaluated by assessing proliferation of co-cultured T_{eff} cells. Cervical cancer infiltrating T_{reg} cells were treated with graded concentrations of ICI with or without E2 and subsequently co-cultured with T_{eff} cells for 5days.*

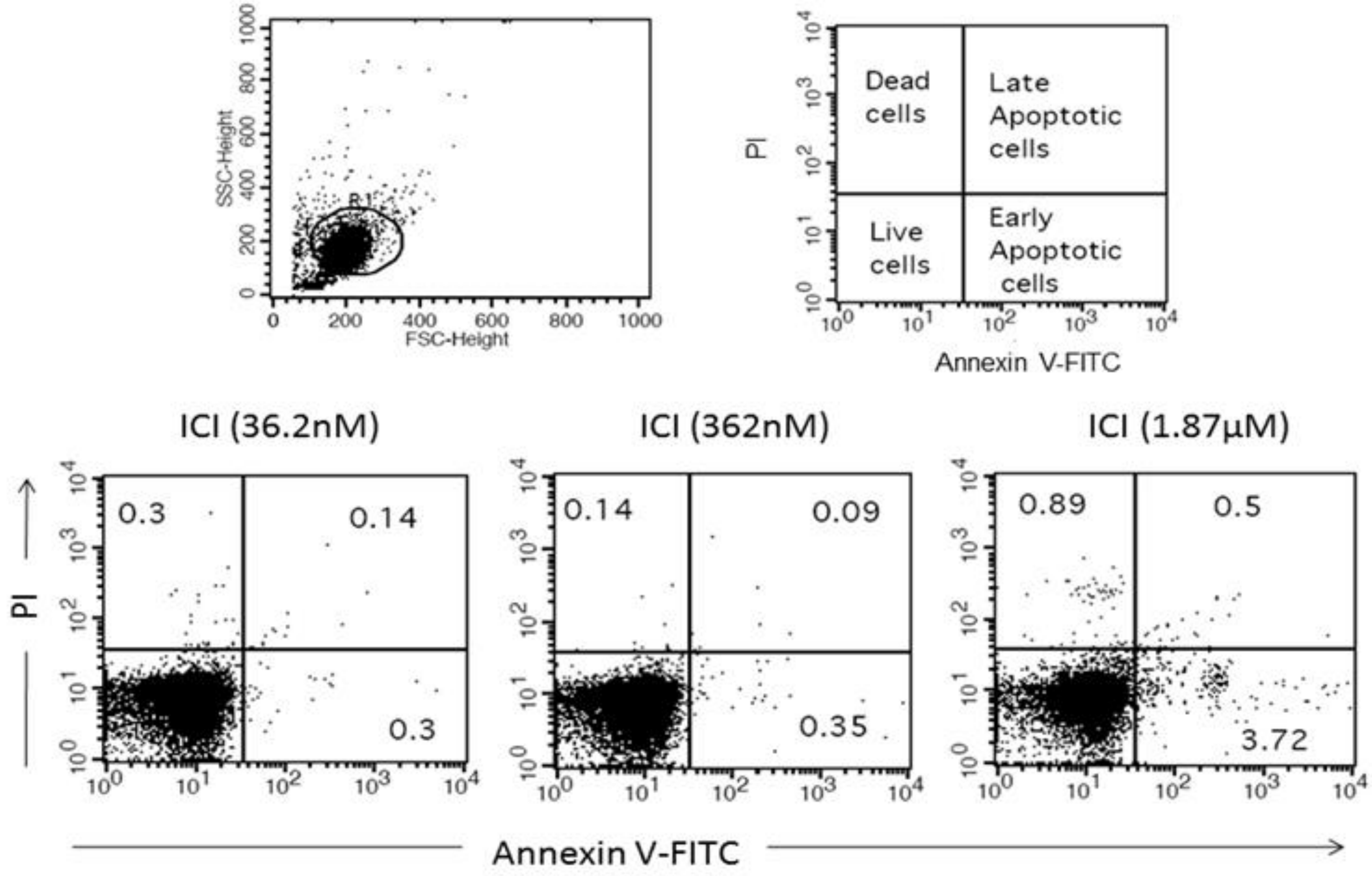

Fig. S16: *T_{reg} cell viability after ICI treatment: MACS-purified and flow-sorted T_{reg} cells were incubated with varying concentrations of ICI for 5 days and their viability assessed using Annexin V and PI staining (n=6).*

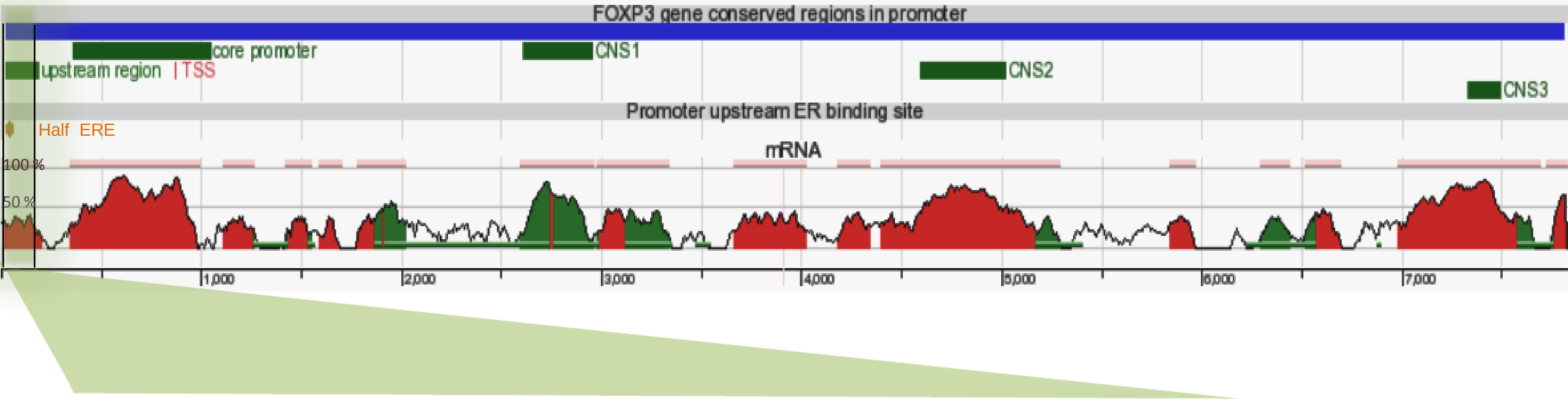

- 943

```
MOUSE                                     -----------------------------------
RAT                                       -----------------------------------
HUMAN                                     ACAAGAGGGTCAGGGTCCCAATGGGCCTCTGGGG
```

```
MOUSE     TTCTTGGTGATGCTGGCTGCATTAAC--------AGCC-ACTGGGGCTGTTCCCAGGTGG
RAT       TTCTTGGAGATGCTGGCTATATTAACATGGGGCCGGCC-GTTAGGGCTGTTCCCAGGTGG
HUMAN     TTCATCGTGAGGATGGATGCATTAATATTGGG--GACCTGCTAGGGACCTTCCCAGTGGG
```

- 846

```
MOUSE     GTGGCTGCTGGGTCAGGGCACTC-AGCAC--AAACATGATGTGGGG-------------
RAT       GTGGCTGCTGGGTCAGAGTGCGCAAGCAC--AAACATAATGTGGAGATACTTGCCTT---
HUMAN     ACAGTGGCTGGGTCAGGGCACTCAAGCCCTAAAACGTGATGAGGCGAGACTTTTCTCTCT
```

```
MOUSE     ---CTCACTCAGAGACTCGCAGCAGCTTCTGGGAGCCAGCCATTCTGAGACTCTCTGATT
RAT       ---CTCACTCAGAGACTCTCAGCAGATTCTGGGAGCCAGCAATTCTGAGACTCTTTGATT
HUMAN     TTCCTCATTCAGTAACTGTCAGTAGATTCTGGGAGCCAGGGATTCTCCGACTCTTCAAGT
```

Primers used:
for Core promoter 3a upstream
for Core promoter 3b upstream

Fig. S17: Alignment maps of human, rat and mouse FOXP3 gene: region upstream of the core promoter. **Underlined mapped pattern represents half ERE located in core promoter upstream region. *Graph : y-axis represent sequence conservation, red colour area is intergenic region and green colour area is rich in simple repeats and transposons*

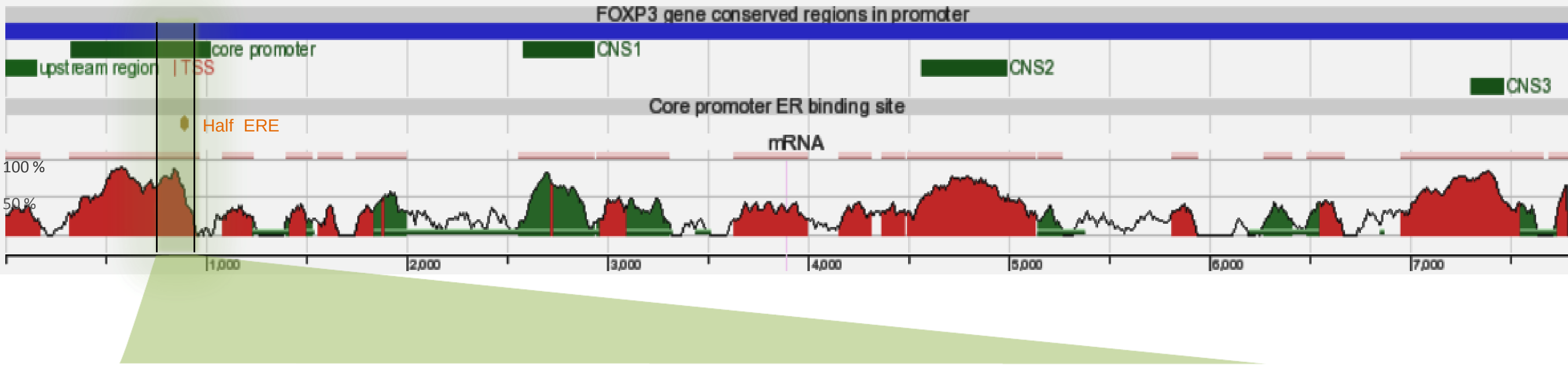


```
MOUSE    CGAGGTCTGCGGCTTCCACGCCGT-------GGTTTTTCTTCTCGGTATAAAAGCAAAGT
RAT      AGAGGTCTGCGGCTTCCACACCGTG------GTTTTTTCTTCTCGGTATAAAAGCAAAGT
HUMAN    AGAGGTCTGCGGCTTCCACACCGTACAGCGTGGTTTTTCTTCTCGGTATAAAAGCAAAGT

                           TSS
MOUSE    TGTTTTTGATAATGTGGCAGTTTCCCACAAGCCAGGCTGATCCCCCTCTAGCAGTCCACT
RAT      TGTTTTTGATAATGTGGCAGTTTCCCACAAGCCAGGCTGATCCCTCTCTGTCAGTCCACT
HUMAN    TTTTTGTGAT-ACGTGACAGTTTCCCACAAGCCAGGCTGATCCTTTTCTGTCAGTCCACT

                + 39
MOUSE    TCACCAAGGTGAGCGAGTGTCCCTGCTCTCCCCCACCAGACAC-AGCTCTGCTGGCGAAA
RAT      TCACTCAGGTGAG----TGTCCCTGCTCTCCCCCACCAGACAC-AGCTCTGCTGGAGAAA
HUMAN    TCACCAAGGTGAG----TGTCCCTGCTCTCCCCTACCAGATGTGGGCCCCATTGGAGGAG

                                   + 114
MOUSE    GTGGCAGAGAGGTATTGAGG--GTGGGTGTCAGGAGCCCACCAGTACAGCTGGAAACACC
RAT      GTGGCAGGGA------------AGGAGTGTCAGGAGCCCTCCAGTACAGCCGGACACACT
HUMAN    ATGGCAGGGAGGTAGGCACGGCGGGGGGTCAGGGGCCCTCTGGTACAGTGGGATGTACC

MOUSE    CAGCCACT----------CCAGGTAAGGACTTTGGAAACTAATACCATTCATCCTAAATG
RAT      ------------------GCAGGTAAGGACTTTTGAAACTAATACCACTCATCCTAAATG
HUMAN    CAGCTACCGTGATTCCAGCCAGGTAAGGTCTTTAAAAA--AATAATAAT-AGAATAAATG
```

Primers used:

for Core promoter 4e downstream

for Core promoter 4d downstream

Fig. S18: Alignment maps of human, rat and mouse FOXP3 gene: core promoter of FOXP3. *Underlined mapped pattern represents a half ERE located in the core promoter 39bp downstream to TSS. *Graph : y-axis represent sequence conservation, red colour area is intergenic region and green colour area is rich in simple repeats and transposons*

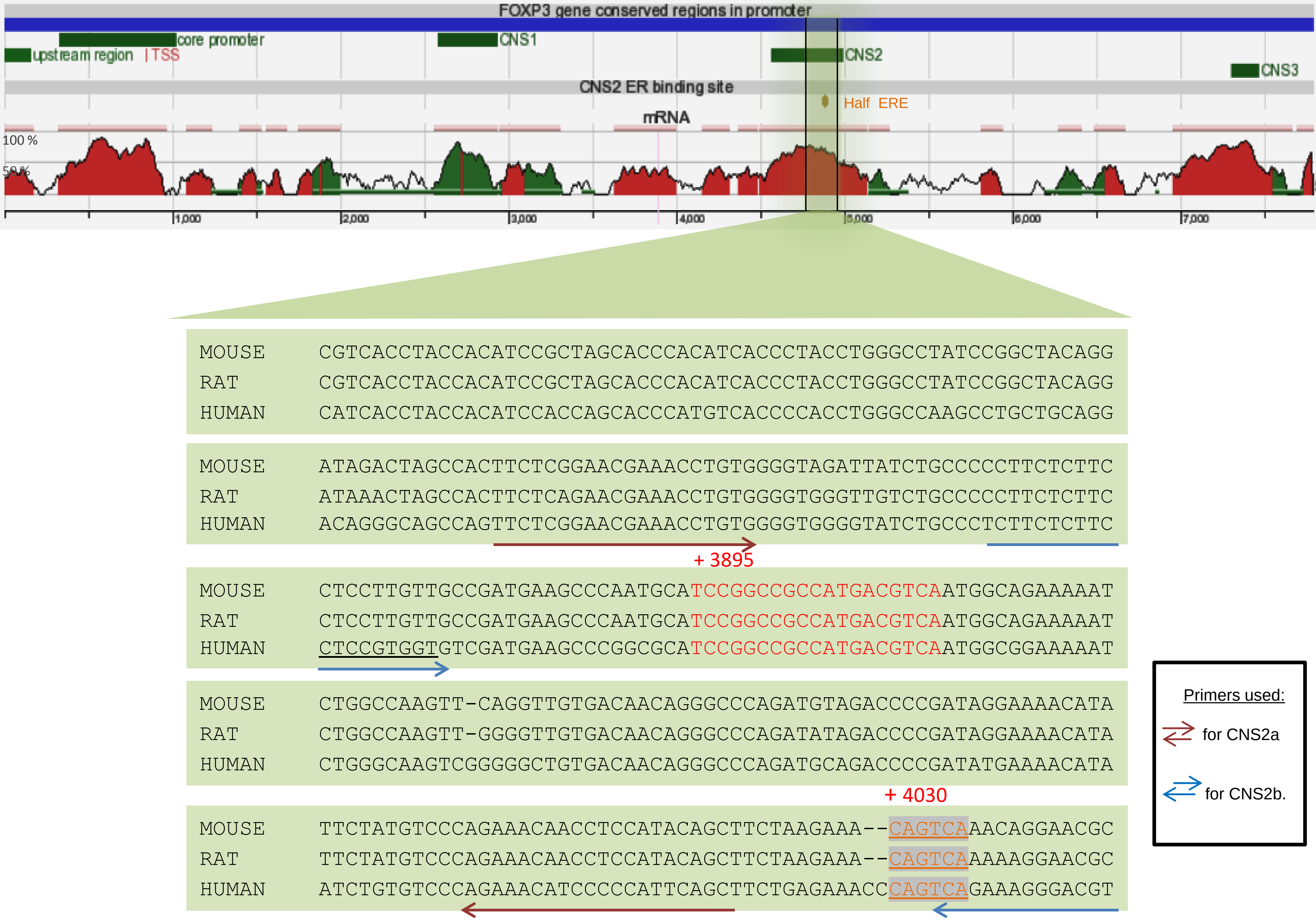

Fig. S19: Alignment maps of human, rat and mouse FOXP3 gene: CNS2 region. *Red font pattern represents full ERE variants in CNS2a; underlined mapped pattern represents half ERE of CNS2b enriched region. *Graph : y-axis represent sequence conservation, red colour area is intergenic region ; green colour indicates region rich in simple repeats and transposons*

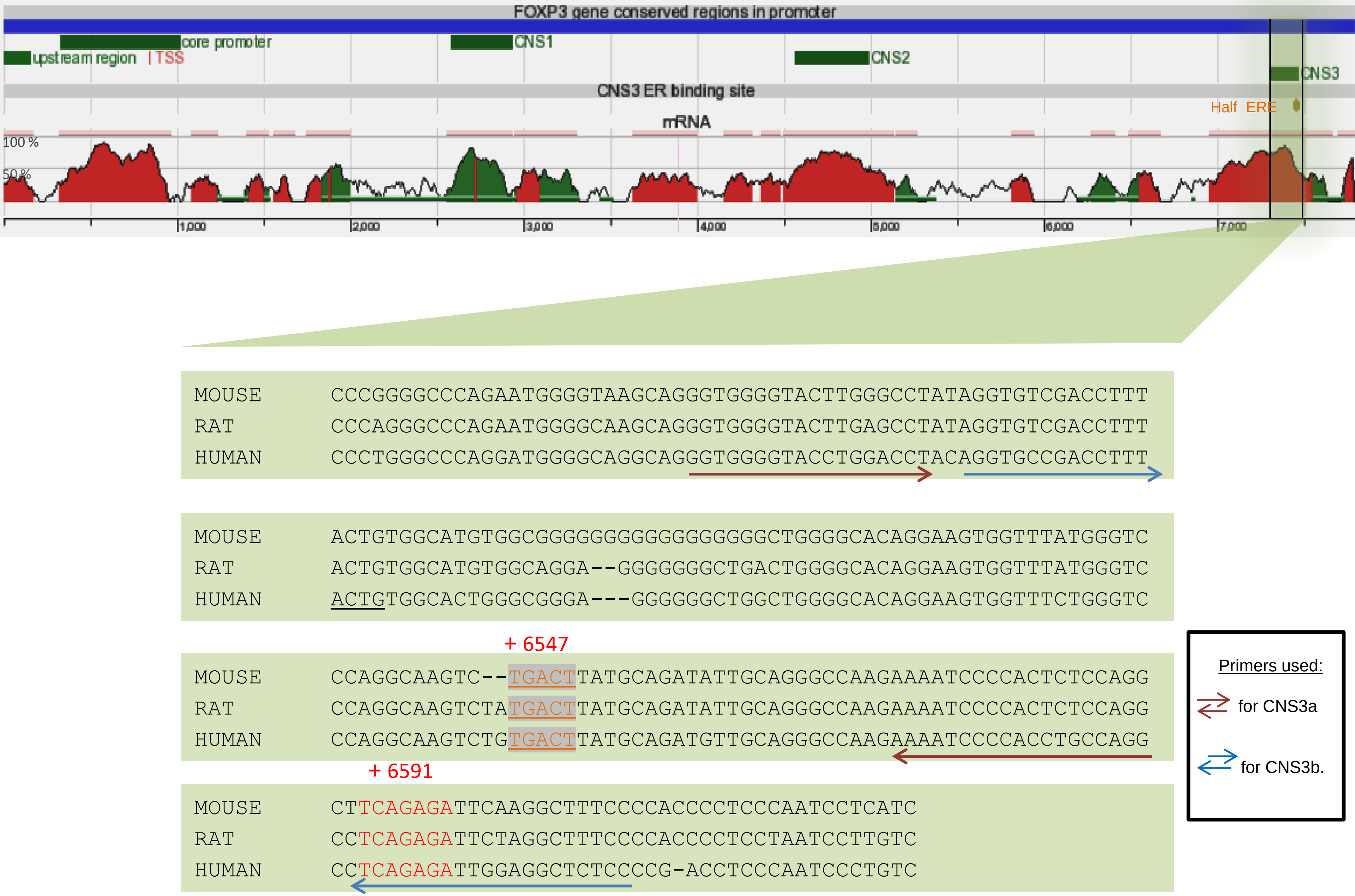

Fig. S20 Alignment maps of human, rat and mouse FOXP3 gene: CNS3 region. ** Underlined mapped pattern represents half ERE of CNS3a enriched region. Red font pattern represent a half ERE in CNS3b enriched region. *Graph : y-axis represent sequence conservation, red colour indicates intergenic region; green colour indicates region rich in simple repeats and transposons.*

ERα 66 kDa

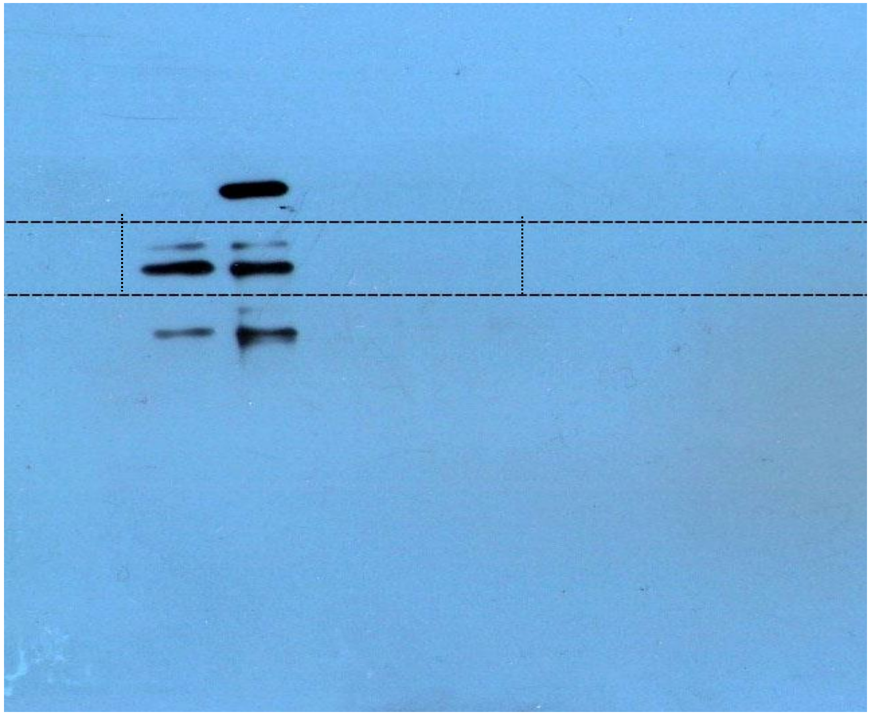

MCF	+	-	-	-	-	-	-	-
Tregs	-	+	+	+	+	+	+	+
ICI 1.8µM	-	-	+	+	-	-	-	-
ICI 362nM	-	-	-	-	+	+	-	-
ICI 36.2nM	-	-	-	-	-	-	+	+
E2 3.6nM	-	-	-	+	-	+	-	+

Fig. S21: *Original blot picture of Fig. 7. IP with anti E2 antibodies and IB with anti ERα antibodies. Dotted lines indicate cropping position.*

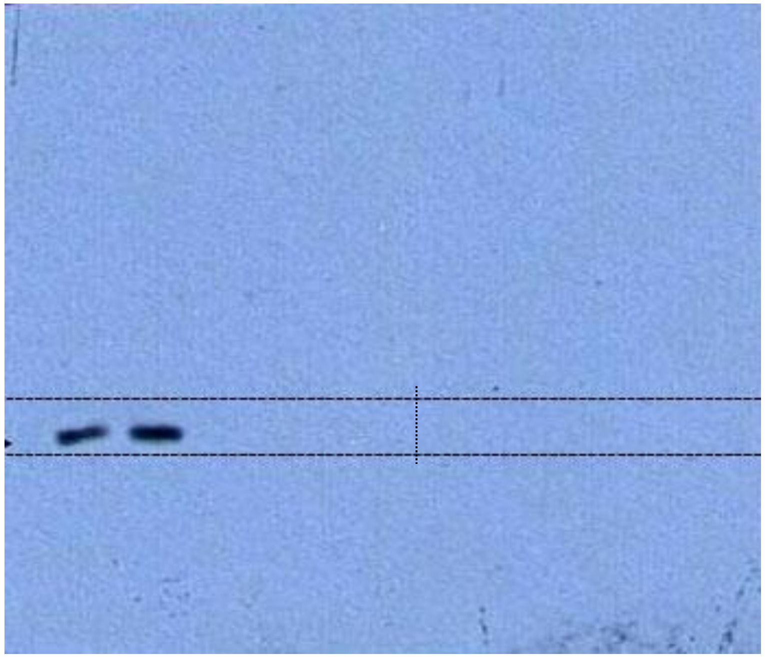

MCF	+	-	-	-	-	-	-	-
Tregs	-	+	+	+	+	+	+	+
ICI 1.8µM	-	-	+	+	-	-	-	-
ICI 362nM	-	-	-	-	+	+	-	-
ICI 36.2nM	-	-	-	-	-	-	+	+
E2 3.6nM	-	-	-	+	-	+	-	+

Fig. S22: *Original blot picture of Fig. 7. IP with anti E2 antibodies and IB with antiFOXP3 antibodies. Dotted lines indicate cropping position.*

β Actin

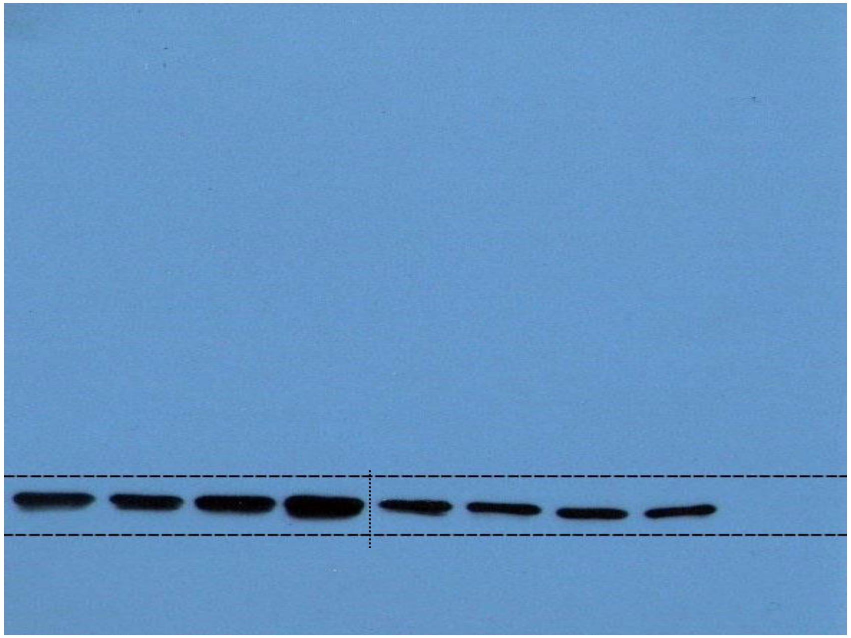

MCF	+	-	-	-	-	-	-	-
Tregs	-	+	+	+	+	+	+	+
ICI 1.8µM	-	-	+	+	-	-	-	-
ICI 362nM	-	-	-	-	+	+	-	-
ICI 36.2nM	-	-	-	-	-	-	+	+
E2 3.6nM	-	-	-	+	-	+	-	+

Fig. S23: *Original blot picture of Fig. 7. IP/IB with beta actin antibodies. Dotted lines indicate cropping position.*

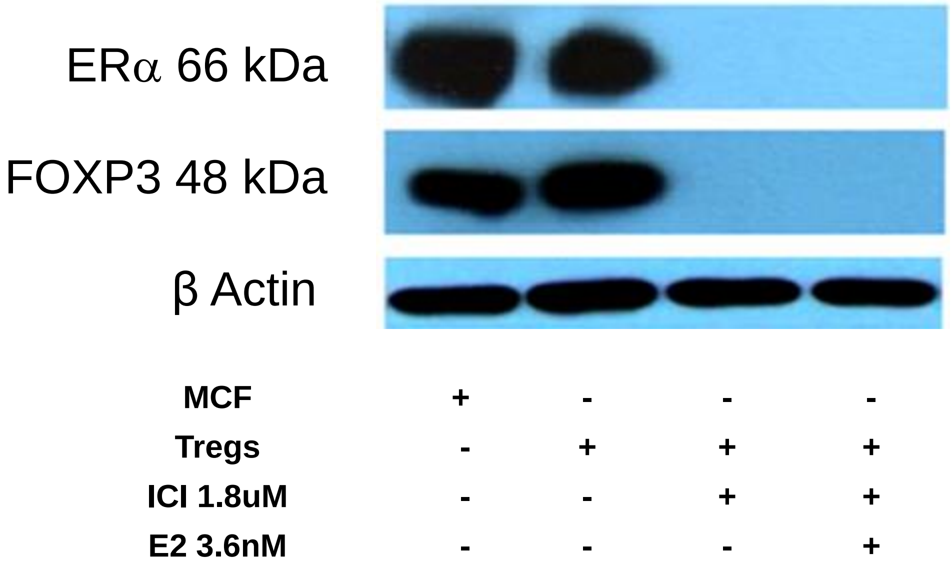

Fig. S24: *FOXP3 co-immunoprecipitation with ERα. Cervical cancer-infiltrating* T_{reg} *cells were treated or not with ICI in the presence or absence of E2 for 72h before lysis. Anti-ERα antibodies were then used to immunoprecipitate protein complexes for immunoblotting with antibodies against ERα and FOXP3. β-actin immunoblots were carried out using lysates pre-immunoprecipitated with anti β-actin antibodies. Data are representative of 6 independent experiments. Gels have been run under the same experimental conditions.*

**Supplementary Table 1**		
**Primer sequences used for real time and RT PCR**		
**Primers**		
**Reverse transcription PCR**		**Sequence**
β Actin	**F Primer**	GAGCGGGAAATCGTGCGTGACATT
	**R Primer**	GAAGGTAGTTTCGTGGATGCC
FOXP3	**F Primer**	ACACCACCCACCACCGCCACT
	**R Primer**	TCGGATGATGCCACAGATGAAGC
ERα	**F Primer**	AAGAGCTGCCAGGCCTGCC
	**R Primer**	TTGGCAGCTCTCATGTCTCC
**Real time PCR**		
GAPDH	**F Primer**	CCACTCCTCCACCTTTGAC
	**R Primer**	ACCCTGTTGCTGTAGCCA
FOXP3	**F Primer**	GCACCTTCCCAAATCCCAGT
	**R Primer**	GGCCACTTGCAGACACCAT
ERα	**F Primer**	TGATTGGTCTCGTCTGGCG
	**R Primer**	CATGCCCTCTACACATTTTCCC

Supplementary Table 2: Sequence of primers used for ChIP qPCR

Primers for ChIP-PCR	Genomic Location Of the amplified region	Forward	Reverse
FOXP3 Promoter Sequence 1	**ChrX:**49268587-49268492	CAGCTCAGCAAACACTGACCTG	CTTCTTGCCCAACCTGACAC
FOXP3 Promoter Sequence 2	**ChrX:**49268364-49268282	CTCCTTTCGCTCTCCCTTT	ATTCCTTCTCCATCCTGGG
FOXP3 Promoter Sequence 3A	**ChrX:**49265643-49265462	AGGGTCAGGGTCCCAATGG	GCCAGGGATTCTCCGACTC
FOXP3 Promoter Sequence 3B	**ChrX:**49265526-49265383	AAAACGTGATGAGGCGAGAC	ACTTACATGCCCCTCACCCT
FOXP3 Promoter Sequence 4A	**ChrX:**49265153-49264976	TCCTCTCACTCACAGAATGGTG	TTCTTCCCCTCACCACAGAG
FOXP3 Promoter Sequence 4B	**ChrX:**49264995-49264872	CTCTGTGGTGAGGGGAAG	CTCTCTCTCTGTGTTTCTCC
FOXP3 Promoter Sequence 4C	**ChrX:**49264891-49264778	GGAGAAACACAGAGAGAGAG	GCAGACCTCTCTCTTCTAAT
FOXP3 Promoter Sequence 4D	**ChrX:**49264833-49264658	TATCAGCGCACACACTCATC	CACCTTGGTGAAGTGGACTG
FOXP3 Promoter Sequence 4E	**ChrX:**49264716-49264598	CGTGACAGTTTCCCACAAG	CTACCTCCCTGCCATCTC
FOXP3 CNS1a	**ChrX:**49263112-49262931	TGCCACAGCTCTTTATTTGG	CCAGAGACAGAAGCCACAGA
FOXP3 CNS1b	**ChrX:**49262950-49262801	TCTGTGGCTTCTGTCTCTGGT	CTGGCTCCAGTACCCACACT
FOXP3 CNS2a	**ChrX:**49260885-49260688	TTCTCGGAACGAAACCTGTG	GCTGAATGGGGGATGTTTCT
FOXP3 CNS2b	**ChrX:**49260848-49260655	TCTTCTCTTCCTCCGTGGT	TGGGACGTCCCTTTCTGA
FOXP3 CNS3a	**ChrX:**49258263-49258114	GGTGGGGTACCTGGACCTA	CTGGCAGGTGGGGATTTT
FOXP3 CNS3b	**ChrX:**49258245-49258091	ACAGGTGCCGACCTTTACTG	GGAGAGCCTCCAATCTCTGA
FKBP6: ERE (neg)	**Chr7:**73332569-73332661	GGGACTCTCGAGGGGATAAG	TGTACCCAAGAACCACGTCA
PS2	**Chr21:**42366775-42366892	ATGGGAGTCTCCTCCAACCT	TTCCGGCCATCTCTCACTAT
BCL11B	**Chr14:**99175272-99175428	TCTCGTGGTGGCGGAAGT	CTCAAGCGCCACATGAAGAC
SETDB1	**Chr1:**150926713-150926817	GGAAAATGGAGGCGCTGA	CGTGGGTACCTAATATCCACAAT

Note: coordinates for UCSC genome version GRCh38/hg38

Supplementary Table 3: Transcription Factor Binding sites on the FOXP3 locus

TFBS on FOXP3

Region	TFBSs	Start position	Detected by using Transfac matrix	Stat. Value P-value < 0.01	RSAT Tool detected score	Detected by experiments	Fuctional evidence	Retrieved from literature (reference)	motif pattern	Conserved motif accross the species
Upstream core promoter	Half ERE	-943	yes	4.00E-004	7.9	chip enriched region in present study			GGTCA	not conserved
	NFKB	-876	yes	8.00E-05	7.6				TAGGGACCTTCCCA	partially conserved
	Half ERE'	-846	yes	4.60E-04	5.6	chip enriched region in present study			GGTCA	conserved in human mouse rat
	ETS1	-797	yes	1.90E-03	5.8				TCCTC	Partially conserved
	STAT5	-769					No functional evidence	Burchill et al, 2007	TTCTGGGAG	conserved in human mouse rat
	STAT5	-753					No functional evidence	Burchill et al, 2007	TTCTCCGACT	Partially conserved
Core promoter	NFKB	-147	yes	5.60E-06			functional evidence	Eckerstorfer et, al, 2010	GAGAACCCCC	conserved in human mouse rat
	RUNX1	-53	yes	2.80E-03			functional evidence	Klunker et al, 2009	CGTGGTT	not conserved
	FOXO3a	-20					functional evidence	Ouyang et al, 2010	GTTGTTTTT	conserved in human mouse rat
	NFKB/NFAT	-1					functional evidence	Barbarulo et al 2011	TTTCCCA	conserved in human mouse rat
	Half ERE'	39	yes	2.8E-03	3.8	chip enriched region in present study			GGTGAG	conserved in human mouse rat
	Sp1	106	yes	1.2E-03	4.4				ACGGCGGGGG	not conserved
	Half ERE'	114	yes	2.6E-03	5.9	chip enriched region in present study			GGGGTCAGGGG	conserved in human mouse rat
	NFKB	121					No functional evidence	Soligo et al, 2011	GGGGCCCTCT	not conserved
	NFKB	139					functional evidence	Soligo et al, 2011	GGGATGTACC	not conserved

Supplementary Table 3 (contd): Transcription Factor Binding sites on the FOXP3 locus

TFBS on FOXP3

Region	TFBSs	Start position	Detected by using Transfac matrix	Stat. Value P-value < 0.01	RSAT Tool detected score	Detected by experiments	Fuctional evidence	Retrieved from literature (reference)	motif pattern	Conserved motif accross the species
CNS2	STAT5	3820					functional evidence	Ogawa et al, 2014	TTCTCGGAA	partially conserved
	RUNX1	3853					functional evidence	Zheng et al, 2010	CTACCACATCC	conserved in human mouse rat
	ETS1	3895					functional evidence	Polansky et al, 2010	TCCGG	conserved in human mouse rat
	Full ERE	3895	yes	0.00097	5.1	chip enriched region in present study			TCCGGCCGCCATGACGTCA	conserved in human mouse rat
	CREB	3906					functional evidence	Kim et al , 2007	TGACGTCA	conserved in human mouse rat
	NFAT	3978					functional evidence	Li et al, 2014	TGAAAACATAATC	conserved in human mouse rat
	FoxO3A	4001					functional evidence	Ouyang et al, 2010	AACATC	partially conserved
	STAT5	4018					functional evidence	Ogawa et al, 2014	TTCTGAGAA	conserved in human mouse rat
	Half ERE*	4030	yes	6.60E-003	3	chip enriched region in present study			CCAGTCA	conserved in human mouse rat
	NFKB	4041					functional evidence	corresponding to KB2 of Long et al, 2009; Li et al, 2014 compared human and mouse FOXP3 CNS2	GGGACGTCCCA	partially conserved
CNS3	RUNX1	6519	yes	3.40E-003	4.2				AGTGGT	conserved in human mouse rat
	SMAD	6536	yes	6.10E-004	5.5				GGCAAGTCT	conserved in human mouse rat
	Half ERE	6547	yes	0.0012	4.8	chip enriched region in present study			TGACTTA	conserved in human mouse rat
	C-Rel	6547					functional evidence	Zheng et al, 2010	AAGAAAATCC	conserved in human mouse rat
	Half ERE*	6591	yes	2.30E-002	1.7	chip enriched region in present study			TCAGAGA	conserved in human mouse rat

*NOTE: Experimentally validated enriched regions motifs in the current study

Criteria* to select : a) Detected by Transfac with p<0.01; b) conserved in 3 species; c) Proven/suggested to be important by other reports; d) in the region experimentally found to be enriched
